# Understanding the functional basis of moral conviction: Is moral conviction related to personal and social identity expression?

**DOI:** 10.1371/journal.pone.0327438

**Published:** 2025-07-03

**Authors:** Lindsay M. Novak, Linda J. Skitka

**Affiliations:** Department of Psychology, University of Illinois Chicago, Chicago, Illinois, United States of America; Pacific Lutheran University, UNITED STATES OF AMERICA

## Abstract

The degree to which one experiences an attitude as a moral conviction is associated with a host of consequences, such as charitable giving, volunteerism, political engagement, resistance to compromise, intolerance of dissenting viewpoints, and acceptance of any means, including violence, to achieve morally preferred ends. Despite these profound ramifications, our understanding of the psychological functions of moral conviction remains limited. In three studies, we tested competing hypotheses about two possible functions of moral conviction: personal identity and social identity expression. Study 1 developed and validated personal and social identity function measures in a U.S. sample and provided an initial test of hypotheses (*N *= 320). Study 2 further validated these measures and tested whether cultural mindset moderated the relationship between identity functions and moral conviction in a U.S. sample (*N* = 364). Study 3 tested hypotheses cross-culturally (i.e., using U.S. and Indian samples, *N* = 300). The personal identity function uniquely predicted moral conviction in all three studies and across six issue domains, whereas the social identity function did not (Studies 1–3). Surprisingly, neither cultural mindset (i.e., an independent and interdependent self-construal or endorsement of the individualizing or binding moral foundations) nor culture moderated these results.

## Introduction

*“…I will no longer be complicit in genocide…I am about to engage in an extreme act of protest…Free Palestine!” – Aaron Bushnell*

*“I am not going to stand up to show pride in a flag for a country that oppresses black people and people of color.” – Colin Kaepernick*

People sometimes engage in acts of moral courage, that is, they stand up in defense of a principle, even when others stand aside [1]. Aaron Bushnell, for example, recently engaged in self-immolation outside the Israeli embassy in Washington, D.C., as an act of protest towards the U.S. involvement in the ongoing Israeli-Palestinian conflict. From the video he live-streamed on Twitch that included his self-immolation, he declared that he could “…no longer be complicit in genocide.” He went on to say, “I am about to engage in an extreme act of protest but compared to what people have been experiencing in Palestine at the hands of their colonizers, it’s not extreme at all” [2]. In other words, Bushnell perceived his moral courage as stemming from his sense of conscience, an aspect of his personal identity [3].

Colin Kaepernick appeared in the media spotlight in 2016 for his gesture of moral courage. He chose to sit or kneel rather than stand during the U.S. National Anthem at professional football games. He explained that “I have to stand up for people who are oppressed” in response to Black people being disproportionately targeted, brutalized, and killed by police. Kaepernick’s actions appear to reflect his social identity as a Black man in America. Bushnell and Kaepernick stood up for their moral convictions, but seemingly for different reasons: personal conscience on the one hand and a need to defend his identity as a Black man on the other.

The present research explores the possible ties between people’s moral convictions about different issues and the personal and social identity functions these moral convictions might serve (Studies 1, 2, and 3) and possible cultural mindset boundaries on these relationships (Studies 2 and 3). Although previous theoretical frameworks have posited the existence of these connections (e.g., [4]), empirical investigations remain scarce, leaving an important gap in knowledge. Researchers who claim that moral convictions serve an identity function or an identity-based reason for holding an attitude have generally inferred rather than directly tested this hypothesis (e.g., [4,5]). In addition, some researchers and theorists claim that moral convictions primarily serve a social identity function (e.g., [5–7]) without considering the possibility that they may serve a personal identity function instead or as well. Finally, moral convictions might serve different identity functions for people as a function of their dominant cultural mindset or orientation. People higher in independent self-construal (i.e., defining themselves in terms of internal attributes and distinctiveness), for example, might use moral convictions to express a sense of personal identity and moral authenticity, whereas people higher in interdependent self-construal (i.e., defining themselves in terms of their connections to other people or groups) might use moral convictions to express their sense of social identity and group belongingness instead.

The goal of the three studies reported here was to address these gaps in knowledge by 1) developing explicit measures of the degree to which people perceive a given attitude as reflecting either a personal or social identity function, 2) establishing that these measures capture distinct constructs and have high internal reliability and validity, 3) testing the degree to which these measures are associated with people’s moral convictions across a diverse set of issues, and 4) testing whether the relationship between these identity functions and moral conviction varies as a function of cultural mindset.

Before describing the specifics of these studies, we first provide a brief review of what we know about the psychology of moral conviction, how attitudes relate to the self, and how needs related to personal identity, social identity, or a combination of both identities may serve as the functional basis of people’s moral convictions.

### Moral conviction

Some but not all of people’s attitudes about specific issues, such as the right to privacy or a commitment to civil rights, are experienced as moral convictions, or one’s feelings or beliefs about morality and immorality, or questions of fundamental right and wrong [8]. Attitudes experienced as moral convictions can be distinguished from attitudes experienced as preferences or matters of convention instead. Attitudes in the domain of preference are experienced as matters of taste or subjective inclination. People are pretty tolerant of others whose tastes are not similar to their own. Someone might prefer that marijuana is legal in their state because they enjoy using it but probably would not end a friendship with someone who disagrees with this position. Conventional attitudes tend to be rooted more in norms or what most people in one’s group happen to believe. Another person, for example, might support legalizing marijuana in their state because legalization has become increasingly normative in the U.S., where many other states have already legalized it. Other people’s positions on marijuana legalization, however, might reflect their moral convictions. Moral convictions tend to be perceived as cultural universals and facts about the world. That X is morally right or wrong is as apparent to a morally convicted perceiver as the idea that 2 + 2 = 4. Someone morally convicted about marijuana legalization will therefore be, among other things, more confident about the correctness of their position and more intolerant of those who do not share it [9–12].

Variance in the degree to which an attitude is perceived as a moral conviction has significant social and political consequences. Stronger moral convictions about a given issue (or candidate) are associated with increased political engagement, such as the likelihood of voting or engaging in activism [13–15]; volunteering [16]; increased inoculation against the usual pressures to conform to group norms or majority influence [17–19]; greater acceptance of deception when it serves the cause [20]; and greater acceptance of unfair procedures or even violence when it helps to achieve morally preferred ends [4,21,22]. The normative implications of these and other findings are both reassuring (moral convictions can protect against obedience to malevolent authorities) and potentially terrifying (moral convictions are associated with rejection of the rule of law and can provide a motivational foundation for violent protest and acts of terrorism).

Although we know a great deal about moral conviction and its consequences, we still know comparatively little about what psychological function or functions moral convictions serve for people. Katz hypothesized that people’s attitudes serve psychological functions, in other words, they serve people’s psychological needs [23]. Although Katz and other theorists outlined several possible needs attitudes could serve (e.g., utilitarian, ego-defensive, knowledge, social-adjustive, and value-expressive), our focus here is on needs related to value expression and social-adjustment. Attitudes that serve a value-expressive function are those that fulfill the need to be consistent with one’s personal values and sense of self as an individual (e.g., [24,25]). In contrast, attitudes that serve a social-adjustive function are those that are shaped by people’s needs to facilitate their social relationships, or that help people interact or gain acceptance from other group members (e.g., [26–28]). Moral convictions may allow people to signal to themselves and others (i.e., express) that they are morally authentic and consistent (i.e., a value expressive function, or what we will refer to as the *personal identity function*), a good group member (a social adjustive-function, or what we will refer to as the *social identity function*), or possibly a combination of these functions or concerns.

We acknowledge that morality is a broad concept and that research on the psychology of morality includes different conceptualizations, such as moral attitudes (i.e., attitudes held with moral conviction), attitude moralization, moral behavior, moral reasoning, and moral judgments (e.g., [29]). Moral conviction studies tend to focus on the antecedents and consequences of people coming to recognize an attitude as being relevant to their sense of morality (e.g., [30–31]; [12]). In the current studies, we are specifically interested in understanding possible psychological needs that might be served by holding attitudes with moral conviction. Although people’s social identities may more strongly predict other aspects of their morality—e.g., moral and blame judgments may be based more on group norms, or people’s larger moral philosophies that may be socially learned—it is more open-ended whether their moral attitudes reflect personal identity, social identity, or combined identity concerns/needs.

### Attitudes and the self

The notion that attitudes can serve an identity function is a long-standing one in social psychology. Sherif and Cantril called these kinds of attitudes “ego-involved,” defined as the “attitudes that the individual identifies with and makes a part of himself (*si*c)” [32] (pp. 126–127). Later theorists referred to attitudes that are more strongly associated with people’s sense of self and identity as attitudes high in centrality [33–35] or as self-defining attitudes [36].

The idea that morally convicted attitudes might serve a self-expressive function (where people’s attitudes may express something about their personal selves or their feelings or concerns about groups important to them) is consistent with research that finds that strength of moral conviction is associated with how central people perceive a given attitude is to their sense of self (e.g., [8]). However, the self is a multi-layered concept, and there is some theoretical debate about which aspect of the self or identity is most closely related to people’s moral convictions. We turn next to hypotheses that argue for the primacy of personal identity versus the primacy of social identity.

### The personal identity hypothesis

Personal identity refers to people’s core, enduring, or “true” self and is the aspect of identity that provides a sense of personal continuity [37,38]. People’s sense of personal identity is the sense of self they wake up to every morning and is “the most enduring and intimate part of the self, that which we most verily seem to be…it is what we think of our ability to argue and discriminate, of our moral sensibility and conscience, of our indomitable will…. ([3], p. 315). Giving up one’s moral beliefs is perceived as akin to no longer being oneself [39,40]. For this reason, people strive to live up to their internalized standards of “ought” and “good” to be morally authentic (e.g., [41,42]). Given the ties between personal identity, conscience, and moral authenticity (e.g., [4,43]), one could reasonably argue that people’s moral convictions primarily serve a personal identity function (the *personal identity hypothesis*).

The hypothesis that moral convictions might primarily serve a personal identity function (rather than a social identity function) is bolstered by research that indicates that strong moral convictions are surprisingly resistant to social influence. For example, in one study, student participants were given an attitude pre-measure at least 24 hours before coming to the lab to participate in the research study. Among other attitude measures, participants reported their support and opposition to using extreme stress techniques (i.e., torture) when interrogating suspected terrorists and the degree to which their position on this issue reflected a personal moral conviction. During the laboratory part of the study, research participants engaged in a computer-mediated group discussion about their support or opposition to the torture of suspected terrorists (an adaptation of the Asch conformity paradigm, [44]). The actual participant always provided their position last after discovering the other “group members” (students at the same university) all had the same position as each other, but a different position on torture from the participant. Participants’ pre-experimental strength of moral conviction about torture uniquely predicted their resistance to majority influence in this context, even when controlling for other indices of attitude strength [17]. This study and others (e.g., [18,19]) reveal that people’s moral convictions are autonomous and relatively uninfluenced by fellow group members, a result that is consistent with the idea that moral convictions have potent ties to personal identity concerns, and comparatively weaker links to their social identity concerns.

Research on the “moral mandate effect” is also more consistent with the hypothesis that moral convictions might be more closely related to people’s sense of personal, rather than their social, identity. The fairness of procedures is generally an important predictor of whether people are willing to accept negative outcomes as nonetheless fair, something known as the “fair process effect” (see [45,46] for reviews). Because people are thought to care more about their relative group standing vis-à-vis authorities and peers than they do about the material outcomes that procedures might yield, they theoretically should be more vested in cues that confirm their status and standing in the group, such as being treated with dignity and respect, than they care about achieving personally desirable outcomes. Consistent with this theoretical perspective that puts more emphasis on people’s social identity concerns and needs, being treated with greater dignity, respect, and propriety not only increases people’s perceptions of procedural fairness but increases their decision acceptance as well—even when outcomes are not otherwise personally desirable (e.g., [47,48]).

Something early work on the fair process effect did not consider, however, was whether people had a moral investment in decision outcomes. Social identity concerns, for example, might be especially strong in shaping people’s perceptions of fairness when non-moral considerations are at stake or when people do not have a clear idea about what the morally correct decision might be. When people have personal moral clarity about what outcome procedures should yield, however, they are more likely to believe that achieving the morally “correct” outcome is more important than achieving it through procedures that enhance the dignity and standing of everyone involved. Indeed, voice and dignity process variables have weak or no effects on people’s perceptions of outcome fairness, decision acceptance, and related variables when people have a strong (rather than weak) moral conviction about the outcome the procedure should achieve (e.g., whether abortion should or should not be legal; see [12,43] for reviews). Researchers interpret the finding that moral concerns trump people’s usual concerns with fair process to mean that people’s personal identity concerns are more important than their social identity concerns when they have a moral stake in decision outcomes (e.g., [4,49,50]). The relative role of personal and social identity concerns in these contexts, however, has been inferred rather than directly measured or manipulated and confirmed.

### The social identity hypothesis

Even if one can mount a case in favor of the *personal identity hypothesis*, there are strong arguments that favor the hypothesis that moral convictions might be more strongly reflective of a social identity function than a personal identity function (the *social identity hypothesis*). One’s social identity is an aspect of the self that reflects one’s membership in a specific group. It incorporates a group’s characteristics, perceptions of the group by oneself and others, and the affective and cognitive aspects that tie the individual to the group (e.g., [51,52]). Many moral theorists, for example, argue that the primary function of morality is to make cooperative life possible (e.g., [53–56]). According to this view, people’s sense of morality is grounded in the shared moral concepts and beliefs of one’s community. Communal moral values function as behavioral imperatives [57–58] and as a standard of human virtue [59,60]. Communal moral values are used to judge whether someone is a good and proper group member [61]. Because violating communal moral values leads to severe consequences (e.g., being excluded from the group), there is intense pressure on group members to internalize and adhere to group norms and standards [62]. For these reasons, people are motivated to seek information about “right” or “wrong” by turning to the groups they see as self-relevant [55], whose beliefs people then internalize into their sense of self [63]. Therefore, people may use moral convictions more to signal to themselves and others that they are valuable members of the group and that they belong, or in other words, to primarily to express their social identity instead of their sense of personal identity.

Consistent with the *social identity hypothesis*, stronger identification with the group mediates the relationship between moral conviction on different political issues and engaging in cause-related collective action efforts, a result that replicates across different politicized issues [5]. In a related vein, perceptions of the group’s morality (and not its competence or sociability) uniquely predicted people’s identification with the group and group pride [64]. Because group identification taps into things such as how much a person sees themselves as a group member or feels strong ties to a group, group identification arguably serves as a proxy for a social identity function. Therefore, there are reasons to anticipate why moral convictions are likely to serve a stronger social identity, compared to personal identity, function instead of the reverse pattern. However, the empirical case for this argument is still inferred, rather than explicitly and directly tested by measuring the degree to which attitudes reflect concerns about people’s social identity.

Of course, the idea that moral convictions might serve a personal versus social identity function need not be zero sum. It could be a case that they serve both functions rather than only one or the other.

### The combined identity hypothesis

Considerable research finds that people rely on one type of identity over the other depending on the salience, context (i.e., issues, beliefs, situations), or other individual differences on the centrality of certain aspects of the self (e.g., [65–69]). For example, personal identity concerns are more salient in individualistic cultural contexts, and social identity concerns are more salient in collectivistic cultural contexts [70].

Even so, it is possible that both personal *and* social identity expression underlie attitudes that are experienced with moral conviction. There may be something psychologically distinct about holding a given attitude for both personal and social identity reasons. Moral convictions may have the consequences they do because they are associated with simultaneous activation of personal *and* social identity concerns. In moral contexts, people may be more likely to see both types of concerns as salient, in a similar fashion to what work on identity fusion might suggest. For example, people with fused identities see their personal and social identities as one, and thus both types of identity can be equally salient and strong motivators in certain contexts (e.g., [71–72]). To explore this possibility, we will test for both additive and interactive effects of personal and social identity functions on moral conviction with the *combined identity hypothesis*.

### Goals of the current studies

The aim of the studies presented here was to test these competing hypotheses, that is, the *personal identity*, *social identity*, and *combined identity hypotheses*. In the current studies, we are interested in whether people perceive their moral attitudes as reflective of concerns or needs related to personal identity, social identity, or both. Study 1 was designed to achieve two things: (1) to develop measures of personal and social identity functions, and (2) to provide a preliminary test of the *personal identity*, *social identity*, and *combined identity* hypotheses. Study 2 was a close and pre-registered replication [https://osf.io/ge57m/?view_only=91c6f3ebcc664f39aba36a16c365e462] of Study 1 that confirmed the factor structure of the personal and social identity function measures and ruled out social desirability as a possible alternative explanation for the results of Study 1. Study 2 also tested whether the perceived association between different identity functions of attitudes and moral conviction varied as a function of individual differences in the cultural orientation of participants in a U.S. sample. Finally, Study 3 was designed to test the generalizability of the results of Studies 1 and 2 across a new set of issues and in different cultural contexts and samples (specifically, the U.S. and India).

## Study 1

In Study 1, we used three different issues—same-sex marriage, gun control (i.e., allowing conceal/carry in Illinois), and capital punishment (i.e., the death penalty)—to test the robustness of the identity function measures and their factor structure and the generalizability of our hypothesis tests. If the personal and social identity functions of attitudes are distinguishable constructs, exploratory factor analyses should yield two-factor solutions across issues; items that measure the degree to which an attitude reflects perceivers’ personal identity function should factor separately from items that reflect their social identity function. Moreover, if these measures capture identity functions of attitudes that generalize across issues, we should observe a similar factor structure across issues.

Once measures of the personal and social identity functions of attitudes are established, we turn our attention to hypothesis testing. If the *personal identity hypothesis* is true, then the degree to which participants perceive a personal identity function of their attitude should explain more unique variance in moral conviction than the degree to which they perceive a social identity function of their attitude. If the *social identity hypothesis* is true, we should see the reverse pattern of results: Participants should perceive a stronger social than personal identity function of morally convicted attitudes. If the *combined identity hypothesis* is true, we should see either an additive or interactive effect of the degree to which participants perceive their attitudes as functionally based on personal versus social identity. The *combined identity hypothesis* would be supported if both constructs contribute unique explained variance in moral conviction (i.e., additive effects), or they synergistically explain more variance than their additive effects do alone (i.e., an interactive effect).

## Method

All studies reported were reviewed and approved by the University of Illinois Chicago institutional review board (Approval #2017−1275). The institutional review board provided waivers of documentation of informed consent and alteration of consent for all studies. Additionally, when studies included minors (i.e., those using college samples), the institutional review board granted a waiver of parental permission as part of the subject pool protocol. Parents were required to sign a blanket parental permission document for a minor to participate in subject pool studies at large, but individual studies did not require parental permission. At the start of each of the current studies, consent was obtained via an online form. Across all studies, we report how we determined our sample size, all data exclusions and data cleaning procedures, and all measures used in the reported studies. Data and analysis code for each study are available at https://osf.io/ge57m/?view_only=91c6f3ebcc664f39aba36a16c365e462. All analyses were conducted in R version 4.2.2 and the following packages were used in the studies: tidyverse [73], psych v 2.2.9 [74], beepr v 1.3 [75], GPArotation [76], relaimpo v 2.2−6 [77], car [78], knitr v 1.42 [79–81], lavaan v 0.6−13 [82], corrplot v 0.92 [83], Hmisc [84], rockchalk v 1.8.157 [85], and pwr v 1.3−0 [86]. Studies 1 and 3 were not preregistered, but Study 2 was preregistered (see https://osf.io/ge57m/?view_only=91c6f3ebcc664f39aba36a16c365e462). Supplemental tables and appendices that are referenced throughout the paper can be found in the supporting information file (S1 File).

### Participants

Three hundred twenty undergraduate students in an introductory psychology course at a large Midwestern university completed the study for partial course credit. We conducted an *a priori* power analysis using the *pwr* package in R to detect a small effect size in a correlational design (*r* = .2) with an α of .05 and 80% power. The results showed that we needed to recruit at least 193 participants. We recruited as many participants as possible during the semester the data was collected (participants were recruited from January 30, 2018 to April 21, 2018), yielding a raw sample of *N* = 354. To clean the data, we removed preview/test responses (*N* = 1), responses with duplicate IDs (including subjects with no ID listed given these indicated incomplete responses; *N* = 31), and those with missing responses for key variables used in the main hypothesis testing analyses (i.e., moral conviction, personal identity, and social identity; *N *= 2), leaving a final sample of *N* = 320.

### Procedure

Participants completed surveys about their attitudes on three issues (same-sex marriage, gun control, and capital punishment). Issues were presented in a randomized order across participants. For each issue, participants indicated whether they supported or opposed the issue and the strength of this position and responded to measures of moral conviction and other attitude strength constructs. Then, participants completed the identity function scale, which asked how much their attitude on an issue reflected items related to personal identity and social identity concerns. After completing measures for the three issues, participants answered demographic questions.

## Measures

### Issue position

To measure issue position, participants first responded to a question of “Do you support or oppose [“legalizing same-sex marriage”/ “allowing conceal/carry in Illinois”/ “capital punishment”]?” with response options of *support, neutral/uncertain,* and *oppose*. Then, participants were branched to one of two questions. For participants who indicated *support* or *oppose*, they responded to the question “How strongly do you [support/oppose] X?” with response options of *slightly, moderately, much,* and *very much*. If participants picked *neutral/uncertain* to the original question, they responded to the question “Do you lean towards supporting or opposing X?” with response options of *lean towards supporting, neutral*, and *lean towards opposing*. These items were combined to form a single bi-polar scale from −5 *strongly oppose* to 5 *strongly support* (with 0 as the neutral point) for each issue.

### Moral conviction

Participants were asked about the degree to which they perceived their attitude on each issue as a reflection of their moral convictions using an established measure (e.g., [87]). Specifically, participants were given the statement “To what extent is your position on X…” and then given two different stems: “…connected to your beliefs about fundamental right and wrong?” and “…a reflection of your core moral beliefs and convictions?” Participants responded on a 5-point scale with point labels of *not at all*, *slightly*, *moderately*, *much*, and *very much*. The two items for moral conviction were reliable (α = .87,.83, and.84 for same-sex marriage, gun control, and capital punishment, respectively). These items were averaged to create a moral conviction score for each issue.

### Attitude strength

Participants indicated how strong their attitudes were on two dimensions: attitude importance and attitude certainty. They were first given the statement “To what extent is your position on X…” and then given two items for attitude importance (“…something that you care a lot about?” and “…personally important to you?”) and two items for attitude certainty (“…something you are certain about?” and “…something you are sure you are correct about?”). Participants responded on a 5-point scale with point labels of *not at all*, *slightly*, *moderately*, *much*, and *very much*. The measures of attitude importance and certainty were reliable (α = .88−.90 and.85−.89, respectively). The two items for each strength indicator were averaged together to create one score for attitude importance and one score for attitude certainty per issue.

### Identity function scales

To measure the degree to which participants’ attitudes on specific issues reflected identity, participants completed measures of personal and social identity functions, which include items adapted from the Aspects of Identity Questionnaire (AIQ-IV; [88]) and attitude function self-report measures (e.g., [89–90]). Participants were asked the general question, “To what extent does your attitude on X reflect the following?” and then were presented with different items related to personal or social identity concerns, as described below. Responses were provided on a 5-point scale with the following point labels: *not at all reflected, slightly reflected, moderately reflected, much reflected,* and *very much reflected.*

**Personal identity function.** The personal identity function measure used the following items: “My sense of who I am as a person,” “My true self,” “The real me,” “My core self,” “Who I am as a person,” “My own personal well-being and self-esteem,” “Important part of who I am,” “My ideas about what kind of person I really am,” “My private opinions of myself,” and “My internal guiding principles” (α = .97 across issues).

**Social identity function**. The social identity function measure consisted of the following items: “How I feel about important others in my life,” “My desire to maintain close relationships,” “My desire to avoid unnecessary conflict with others,” “A signal to others that I am a good group member,” “Values of the group of people most important to me,” “My identification with central groups in my life,” “My desire to avoid being rejected by others who are important to me,” “Reputation (what others think of me),” “Feelings of connectedness with those I am close to,” “Relationships with those I feel close to,” and “External factors that guide my principles and values” (α = .94−.96 across issues).

### Attention checks

Three attention checks were included in the study. The first attention check was included with the same-sex marriage set of questions, and stated, “If you read this question, please select ‘much reflected’”. The second check was included in the gun control questions and stated, “If you read this question, select ‘Not at all reflected’”. The third attention check was included in the capital punishment block of questions and stated, “If you read this question, please select ‘slightly reflected’”.

## Results

The results for Study 1 are organized into two sections. The first section reports on scale development and measurement of the personal and social identity function scales. The second section reports on hypothesis tests.

### Scale development

We first conducted separate exploratory factor analyses (EFA) for each issue using oblimin rotation and Principal Axis Factoring (PAF) extractions. These analyses yielded similar two-factor solutions across all three issues (see S1 Table in S1 File for factor loadings using oblimin rotation and S2 Table in S1 File using a varimax rotation – similar factor loadings were found regardless). The personal and social identity function items loaded primarily on the first and second factors, respectively, with two exceptions. The items *“How I feel about important others in my life”* and *“External factors that guide my principles and values”* unexpectedly loaded better on the personal, rather than the social, identity measure for one issue. We therefore excluded these items. Scales using the average of the items retained for the personal and social identity function scales were highly reliable across issues (α = .94−.97). Taken together, the results yielded the expected two-factor solution, capturing the degree to which participants’ attitudes reflected personal identity and social identity functions, respectively.

### Hypothesis testing

Table 1 summarizes descriptive statistics and correlations. People perceived that their attitudes more strongly reflected a personal identity function than a social identity function across all three issues [*t*(319) = 4.69, *p* < .001 for same-sex marriage, *t*(319) = 6.00, *p* < .001 for gun control, and *t*(319) = 8.59, *p* < .001 for capital punishment]. It should be noted that the means for the personal and social identity functions of attitudes were below the scale midpoints for each issue, a result that suggests that, on average, participants did not perceive their attitudes to be strongly held for personal or social identity reasons. Consistent with the hypothesis that strength of moral conviction would be related to personal identity, social identity, or both identity functions, personal and social identity functions were both positively correlated with moral conviction across all three issues. As perceptions of the personal or social identity functions of attitudes increased, the strength of moral conviction for those attitudes also increased. The identity functions were also positively correlated across all three issues. The more strongly participants perceived an attitude expressed their personal identity, the more strongly they also perceived the same attitude expressed their social identity (and vice versa). These correlations ranged from .61 to .65 across issues, suggesting they share about 30% variance. Although there is some overlap, these results are consistent with the conclusion that the constructs are conceptually distinct.

**Table 1:** Descriptives and correlations for key analytic variables across issues in Study 1.

*Issue*	*Variable*	*M*	*SD*	1	2
Same-sex Marriage	1) Moral conviction	3.39	1.33		
	2) Personal identity	2.77	1.33	.65**	
	3) Social identity	2.48	1.17	.43**	.62**
Gun Control	1) Moral conviction	3.12	1.18		
	2) Personal identity	2.61	1.19	.63**	
	3) Social identity	2.28	1.10	.40**	.63**
Capital Punishment	1) Moral conviction	2.97	1.16		
	2) Personal identity	2.44	1.15	.62**	
	3) Social identity	1.98	1.03	.39**	.61**

*N* = 320 (using complete pairwise observations).

*p < .05. **p < .01.

To put the *personal identity*, *social identity*, and *combined identity hypotheses* to stronger tests, hierarchical regressions were conducted with the predictors of personal and social identity functions in the first step, and the personal and social identity function interaction in the second step, to predict strength of moral conviction. As can be seen in Table 2, strength of the personal identity function of an attitude uniquely predicted moral conviction, whereas strength of the social identity function of an attitude did not, a result that replicated across all three issues (findings at odds with the *social identity hypothesis* and the additive version of the *combined identity hypothesis)*.

**Table 2:** Hierarchical regression analyses (standardized coefficients) predicting moral conviction across issues in Study 1.

	*Same-sex marriage*				*Gun control*				*Capital punishment*			
Predictor	*β*	*SE β*	*LL*	*UL*	*β*	*SE β*	*LL*	*UL*	*β*	SE *β*	LL	UL
Step 1												
Personal identity	.63**	.05	.52	.74	.62**	.06	.51	.73	.61**	.06	.50	.72
Social identity	.03	.05	−.07	.14	<.01	.06	−.11	.12	.02	.06	−.09	.13
*R* ^*2*^_*adj*_	.42				.39				.38			
Step 2												
Personal identity*Social identity	−.07	.04	−.15	.01	−.09*	.05	−.18	−.01	−.05	.04	−.14	.04
*R* ^*2*^_*adj*_	.42				.39				.38			
*ΔR* ^*2*^_*adj*_	.00				.00				.00			

*Note*. *N *= 320 (using complete pairwise observations). LL = lower limit for 95% CI for β; UL = upper limit for 95% CI for β.

*p < .05. **p < .01

The interactions between the perceived personal and social identity functions of an attitude were either not significant (for the issues of same-sex marriage and capital punishment) or not meaningful (i.e., for the issue of gun control, the personal identity x social identity function interaction did not account for more than 1% of the total *R*^2^ for that step in the model), a result that was inconsistent with a synergistic version of the *combined identity hypothesis.* Analyses controlling for attitude strength indices (i.e., importance and certainty) and analyses that applied data exclusions led to similar conclusions (see S3 and S4 Tables in S1 File). In sum, the results of Study 1 supported the *personal identity hypothesis* and were inconsistent with the *social identity* and *combined identity hypotheses*.

## Discussion

The results of Study 1 supported a two-factor solution of the identity functions measure. Personal and social identity functions of attitudes were distinguishable and measurable concepts, a result that replicated across all three issues. The results supported the *personal identity hypothesis*; the more an attitude was perceived as reflective of one’s personal identity, the more the same attitude was perceived as a moral conviction. In contrast, the social identity function did not explain any unique variance in moral conviction, a result inconsistent with the *social identity hypothesis* and the additive version of the *combined identity hypothesis*. Finally, the perceived personal and social identity functions of an attitude did not interactively predict moral conviction at meaningful levels, a result at odds with the synergistic version of the *combined identity hypothesis*.

One major strength of Study 1 was the inclusion of robustness checks to establish that the factor structure of the personal and social identity function measures and tests of hypotheses replicated across issues. The conclusion in favor of the *personal identity hypothesis* was replicated across all issues studied.

## Study 2

The goals of Study 2 were to replicate Study 1 and to explore possible boundary conditions on the conclusion that moral convictions more strongly reflected people’s personal identity than their social identity concerns. The observation in Study 1 that the personal identity function was a stronger predictor of people’s moral convictions about the same issue is open to several interpretations. One interpretation is that moral convictions have a stronger connection to people’s sense of personal identity and moral authenticity than to their sense of social identity and embeddedness in important social groups. Other possibilities, however, are that our choice of sample—an undergraduate sample of American adults—may have stacked the deck in favor of the *personal identity hypothesis*, either because of strong social desirability pressures that may have encouraged people to respond by reporting a more personal than social identity function for their attitudes, or because Americans on average have more individualistic (or independent) than collectivistic (or interdependent) conceptions of self [70].

People with a more individualistic mindset (i.e., independent self-construal) tend to emphasize their personal attributes, values, and achievements over more interdependent aspects of self. In contrast, people with interdependent self-views emphasize seeing themselves in the context of others and as part of their groups and relationships with others, including the characteristics they share with other group members and others’ achievements, over personal attributes, values, or achievements [70,91,92]. There are important differences in the degree to which individuals within different cultures emphasize whether they see themselves primarily through one or another of these lenses. More individualistic cultures tend to have individuals who adopt an independent self-construal, whereas individuals living in more collectivistic cultures tend to have more interdependent self-construal. That said, there is considerable within-culture variation in which perception of self—the self as an individual, or the self as embedded in the group—people adopt as their primary sense of self, including within the United States [93,94].

Thus, there may be important individual differences in the degree to which people perceive their attitudes as reflecting these different aspects of self as well. People who adopt a stronger independent mindset should be more likely to hold attitudes that reflect their sense of personal identity than their social identity. Conversely, people who adopt a stronger interdependent mindset should be more likely to base their attitudes on their social identity rather than their personal identity (the *mindset hypothesis*). To capture these possibilities, Study 2 was designed to rule out social desirability as an alternative explanation for the results of Study 1, and to test whether within-culture differences in cultural mindsets moderate the degree to which people perceive a personal or a social identity function of their moral convictions.

We also tested hypotheses using two different operationalizations of cultural mindset. Independent and interdependent self-construals are commonly measured using self-construal measures (e.g., [95,96]). However, because we are testing hypotheses about people’s moral convictions, it might be more appropriate to use measures that assess the degree to which people’s moral worldviews are more independently or interdependently oriented. Research has revealed two primary foundational concerns that shape people’s sense of morality: The individualizing foundations (that emphasize fairness and harm) and the binding foundations (that emphasize aspects of living in groups, such as respect for authority, the importance of loyalty to the ingroup, and purity; see [97–99]). If the *mindset hypothesis* is true, we predict that people who more strongly endorse the individualizing moral foundations should be more likely to base their moral convictions on a personal identity function, whereas those who more strongly endorse the binding moral foundations should be more likely to base their moral convictions on a social identity function.

In summary, the goals of Study 2 were to (1) confirm the factor structure of the personal and social identity function scales in a new sample, (2) test the replicability of the finding that moral conviction is related more strongly to a personal than social identity function, (3) test a possible boundary condition on the Study 1 results supporting the *personal identity hypothesis*, specifically, whether cultural mindset moderates the relationship between (social and/or personal) identity functions and moral conviction, and finally (4) test additional robustness checks, such as whether controlling for socially desirable responding changes any of our conclusions.

## Method

### Participants

Three hundred sixty-four students in an introductory psychology course at a large Midwestern university completed both Time 1 and Time 2 measures for partial course credit. We conducted an *a priori* power analysis using the *pwr* package in R to determine the sample size needed to detect a small effect of *r* = .18−.20 (corresponding to an *f*^*2*^ of .035−.05) at 80% power with an alpha level of .05 and the 11 predictors included in the final step of the regression model (i.e., main effect and interaction terms). The *r* value was a conservative estimate based on the size of the relationship between each type of identity and moral conviction from Study 1. The power analysis revealed that 478 participants would be needed to get an effect of *r *= .18. In our preregistration for this study, we stated that for our sample size, we either a) aimed to collect *N* = 500 participants, anticipating that some people would not complete all parts of the task and that some would not pass attention checks, or b) collect however many participants we could in a semester before it ended, whichever came first. We recruited participants for Time 1 of the study from February 12, 2020 to May 1, 2020 and participants for Time 2 from February 14, 2020 to May 1, 2020. Data collection stopped due to the semester ending. After removing preview/test responses (*N *= 7 for Time 1 and *N *= 9 for Time 2), removing duplicate IDs (including responses with no ID listed given this meant that participants had incomplete responses; *N* = 51 for Time 1 and *N* = 16 for Time 2), and removing missing responses on key main analysis variables (*N* = 3), we had a final combined sample size of *N* = 364. The observed power with the final sample size was 84% power to detect an effect size of *f*^*2*^ = .05 (equivalent to *r* = .20).

Participants in the sample were predominantly female (62.57%), with a mean age of 19.23 years old (*SD* = 1.66). The sample was ethnically diverse, largely consisting of Latino/a (39.01%) and Asian American (25.55%) individuals, but also included White (19.51%) and Black individuals (7.42%). Participants varied in their generational status, with largely first-generation (38.90%) and second-generation immigrants (44.38%) in the sample, followed by third-and-higher generation immigrants (16.71%). Participants also spoke languages at home other than English (51.92% Non-English monolingual) or were bilingual (18.68% English + second language). Given the demographic diversity of the sample, we had confidence that we would also have more diversity in cultural mindsets (e.g., interdependent mindset) than a college sample might typically have.

### Procedure

Participants completed the Moral Foundations Questionnaire (MFQ), self-construal scale, and social desirability scale at Time 1 of the study. At Time 2 (at least 24 hours after the Time 1 session), the same procedure as Study 1 was used except for measuring some additional demographic variables (e.g., immigrant status).

## Measures

### Time 1 measures

Participants completed individual difference measures of MFQ, self-construal, and social desirability at Time 1. The primary operationalization of individualistic versus collectivistic mindsets was the *individualizing* and *binding* moral foundations [98], respectively. The secondary operationalization for individualistic versus collectivistic mindsets was the interdependent and independent self-construal subscales [95]. This scale served as a back-up operationalization to examine the quality of using *individualizing* versus *binding* foundations as an operationalization for general cultural mindset. Using self-construal subscales as predictors should produce results similar to those of using the Moral Foundations Questionnaire if the foundations are a good proxy for cultural mindset.

**Moral Foundations Questionnaire.** We used the Moral Foundations Questionnaire to capture individual differences in cultural mindsets [98]. The questionnaire has two parts. In Part 1, participants were asked: “When you decide whether something is right or wrong, to what extent are the following considerations relevant to your thinking? Please rate each statement using this scale.” Participants rated these items on a 6-point scale, with point labels of *not at all relevant (This consideration has nothing to do with my judgments of right and wrong), not very relevant, slightly relevant, somewhat relevant, very relevant,* and *extremely relevant (This is one of the most important factors when I judge right and wrong)*. Part 2 instructed participants to read statements and indicate how much they agreed or disagreed with them (*1 strongly disagree* to *6 strongly agree* with no midpoint).

***Individualizing Foundations.*** For Part 1, we asked how morally relevant each of the following were for participants with the following items: “Whether or not someone suffered emotionally,” “Whether or not someone cared for someone weak or vulnerable,” “Whether or not someone was cruel,” “Whether or not some people were treated differently than others,” “Whether or not someone acted unfairly,” and “Whether or not someone was denied his or her rights.” For Part 2, participants indicated how much they agreed or disagreed with the following: “Compassion for those who are suffering is the most crucial virtue,” “One of the worst things a person could do is hurt a defenseless animal,” “It can never be right to kill a human being,” “When the government makes laws, the number one principle should be ensuring that everyone is treated fairly,” “Justice is the most important requirement for a society,” and “I think it’s morally wrong that rich children inherit a lot of money while poor children inherit nothing.” Responses to these items were averaged to measure the individualizing foundations (α = .81).

***Binding Foundations.*** In Part 1, participants responded to the degree to which they endorsed the following items as morally relevant —“Whether or not someone’s action showed love for his or her country,” Whether or not someone did something to betray his or her group,” “Whether or not someone showed a lack of loyalty,” “Whether or not someone showed a lack of respect for authority,” “Whether or not an action caused chaos or disorder,” “Whether or not someone violated standards of purity and decency,” “Whether or not someone did something disgusting,” and “Whether or not someone acted in a way that God would approve of”. In Part 2, we measured the degree to which participants agreed or disagree with a series of statements: “I am proud of my country’s history,” “People should be loyal to their family members, even when they have done something wrong,” “It is more important to be a team player than to express oneself,” “Respect for authority is something all children need to learn,” “Men and women each have different roles to play in society,” “If I were a soldier and disagreed with my commanding officer’s orders, I would obey anyway because that is my duty,” “People should not do things that are disgusting, even if no one is harmed,” “I would call some acts wrong on the grounds that they are unnatural,” and “Chastity is an important and valuable virtue.” Items were averaged together to make a single binding foundations score for each participant (α = .87). It should be noted that the item “Whether or not someone conformed to the traditions of society” from the authority subscale was not included due to a programming error.

**Self-construal.** Self-construal was measured with two subscales: independent and interdependent. Participants responded to the following statements on a 7-point scale of *1 strongly disagree* to *7 strongly agree* [95].

***Independent Self-construal.*** “I’d rather say “No” directly, than risk being misunderstood,” “Speaking up during a class is not a problem for me,” “Having a lively imagination is important to me,” “I am comfortable with being singled out for praise or rewards,” “I act the same way at home that I do at school (or work),” “Being able to take care of myself is a primary concern for me,” “I act the same way no matter who I am with,” “I can talk openly with a person who I meet for the first time, even when this person is much older than I am,” “I prefer to be direct and forthright when dealing with people I’ve just met,” “I enjoy being unique and different from others in many respects,” “My personal identity, independent of others, is very important to me,” “I do my own thing, regardless of what others think,” “I feel it is important for me to act as an independent person,” “I try to do what is best for me, regardless of how that might affect others,” and “I value being in good health above everything”. Items were averaged together to get a composite score (α = .83).

***Interdependent Self-construal.*** “I have respect for the authority figures with whom I interact,” “It is important for me to maintain harmony within my group,” “My happiness depends on the happiness of those around me,” “I would offer my seat on a bus to my professor,” “I respect people who are modest about themselves,” “I will sacrifice my self-interest for the benefit of the group I am in,” “I often have the feeling that my relationships with others are more important than my own accomplishments,” “I should take into consideration my parents’ advice when making education/career plans,” “It is important to me to respect decisions made by the group,” “I will stay in a group if they need me, even when I’m not happy with the group,” “If my brother or sister falls, I feel responsible,” “I feel my fate is intertwined with the fate of those around me,” “I feel good when I cooperate with others,” “I usually go along with what others want to do, even when I would rather do something different,” and “Even when I strongly disagree with group members, I avoid an argument”. Items were averaged together to get a final composite score (α = .80).

**Social desirability.** Social desirability was measured with a short-form version of the Marlowe-Crowne scale that has 13 items [100]. Items were scored on a true-false scale. Participants were instructed to “Read each item and decide whether it is true (T) or false (F) for you. Try to work rapidly and answer each question by selecting the T or the F.

The following items were used: “It is sometimes hard for me to go on with my work if I am not encouraged,” “I sometimes feel resentful when I don’t get my way,” “On a few occasions, I have given up doing something because I thought too little of my ability,” “There have been times when I felt like rebelling against people in authority even though I knew they were right,” “No matter who I’m talking to, I’m always a good listener,” “There have been occasions when I took advantage of someone,” “I’m always willing to admit when I make a mistake,” “I sometimes try to get even rather than forgive and forget,” “I am always courteous, even to people who are disagreeable,” “I have never been irked when people expressed ideas very different from my own,” “There have been times when I was quite jealous of the good fortune of others,” “I am sometimes irritated by people who ask favors of me,” and “I have never deliberately said something that hurt someone’s feelings.” Higher levels of socially desirable responding were assessed by a count of the “false” responses (α = .62).

**Attention checks.** Three attention check items were included in the Time 1 measures, but we only preregistered the open-ended item as the one to be used in exclusion analyses. This attention check item was at the end of the survey, where participants were asked the following: “What is the topic of this study? – Sometimes participants do not carefully read the instructions. To correctly answer this question, please select the option “other” and write down the name of your favorite movie.” The answer choices were values, Shopping preferences, I don’t remember, and Other (please specify:__________).

### Time 2 Measures

Measures of issue position, moral conviction (α = .82−.83 across issues), attitude importance (α = .84−.89 across issues), and attitude certainty (α = .87−.90 across issues) were measured using the same items as Study 1.

#### Identity function scale.

The personal and social identity function items were those used in the hypothesis testing analyses in Study 1 (i.e., the items left from the factor analysis). The personal identity function scale had an α = .96 across issues and the social identity function subscale had an α = .91−.95 across issues.

#### Attention checks.

For Time 2 of the study, the attention check item that was preregistered for exclusion analyses was given at the end, where participants were asked the following: “What is the topic of this study? Sometimes participants do not carefully read the instructions. To correctly answer this question, please select the option “other” and write down the name of your favorite food.” The answer choices were Political issues, Shopping preference, I don’t remember, and Other (please specify:__________).

## Results

We present the results in the following order: confirmatory factor analyses, descriptive statistics, replication analyses, tests of possible interactions, and a summary of robustness checks.

### Confirmatory analyses

Our first goal was to test whether the identity function measures had the expected two-factor structure using confirmatory factor analysis (CFA). A two-factor model, with the personal identity function items on one factor and the social identity function items on another, fit the data better than a one-factor model across all three issues (see S5 Table in S1 File for the fit indices and S6 Table in S1 File for the factor loadings for the two-factor models per issue). These results therefore confirm the identity factors of personal identity and social identity functions.

### Descriptive statistics

Similar to Study 1, participants perceived their attitudes to more strongly reflect their personal than their social identity, *t*(363) = 4.42, *p* < .001 for same-sex marriage, *t*(363) = 7.33, *p* < .001 for gun control, and *t*(363) = 8.65, *p* < .001 for capital punishment, but all means for the identity scales were below the midpoint (see Tables 3–5). On average, participants reported more agreement with the individualizing (*M* = 3.75, *SD* = 0.69) than binding (*M* = 2.93, *SD* = 0.73) moral foundations, *t*(363) = 19.09, *p* < .001, and reported stronger independent (*M* = 5.00, *SD* = 0.81) than interdependent (*M* = 4.83, *SD* = 0.75) self-construal, *t*(365) = 3.68, *p* < .001. The personal and social identity functions were both positively correlated with moral conviction across all three issues, such that perceiving greater personal or social identity concerns in a particular attitude was associated with greater moral conviction for the same attitude. Additionally, both identity functions were positively associated with each other, meaning that the more an attitude was reflective of personal identity, the more reflective the same attitude was of social identity.

**Table 3:** Descriptive statistics and correlations for the issue of same-sex marriage in Study 2.

*Variable*	*M*	*SD*	1	2	3	4	5	6	7
1. Moral conviction	3.45	1.28							
2. Personal identity	2.78	1.24	.51**						
3. Social identity	2.52	1.05	.32**	.53**					
4. Binding	2.92	0.73	−.02	<−.01	.14**				
5. Individualizing	3.75	0.69	.27**	.23**	.28**	.33**			
6. Independent SC	5.00	0.81	.03	−.04	.03	.32**	.26**		
7. Interdependent SC	4.83	0.75	−.04	<.01	.10	.38**	.20**	.35**	
8. Social desirability	6.34	2.56	−.05	−.03	<−.01	.16**	.06	.14**	.05

*Note*. *N* = 364 (using complete pairwise observations). SC = self-construal.

*p < .05. **p < .01.

**Table 4:** Descriptive statistics and correlations for the issue of gun control in Study 2.

*Variable*	*M*	*SD*	1	2	3	4	5	6	7
1. Moral conviction	3.19	1.14							
2. Personal identity	2.66	1.14	.65**						
3. Social identity	2.29	1.01	.42**	.61**					
4. Binding	2.92	0.73	.06	.14*	.28**				
5. Individualizing	3.75	0.69	.20**	.15**	.11*	.33**			
6. Independent SC	5.00	0.81	.12*	.07	.08	.32**	.26**		
7. Interdependent SC	4.83	0.75	.07	.05	.11*	.38**	.20**	.35**	
8. Social desirability	6.34	2.56	−.05	.04	.04	.16**	.06	.14**	.05

*Note*. *N* = 364 (using complete pairwise observations). SC = self-construal.

*p < .05. **p < .01.

**Table 5:** Descriptive statistics and correlations for the issue of capital punishment in Study 2.

*Variable*	*M*	*SD*	1	2	3	4	5	6	7
1. Moral conviction	3.22	1.11							
2. Personal identity	2.51	1.12	.55**						
3. Social identity	2.05	1.03	.31**	.56**					
4. Binding	2.92	0.73	<.01	.15**	.30**				
5. Individualizing	3.75	0.69	.13*	.09	.07	.33**			
6. Independent SC	5.00	0.81	.09	.10*	.05	.32**	.26**		
7. Interdependent SC	4.83	0.75	<.01	.11*	.13*	.38**	.20**	.35**	
8. Social desirability	6.34	2.56	−.03	.07	.07	.16**	.06	.14**	.05

*Note*. *N* = 364 (using complete pairwise observations). SC = self-construal.

*p < .05. **p < .01.

The correlations between the identity function measures and the different operationalizations of cultural mindsets were inconsistent across issues. Endorsement of the personal identity function of attitudes was positively associated with endorsement of the individualizing moral foundations for two of the three issues, with the binding foundations for two issues, and with an independent self-construal and interdependent self-construal for one of three issues. Endorsement of a social identity function of attitudes was positively related to the binding foundations for all issues, with individualizing moral foundations for two of the three issues, an interdependent self-construal for two of three issues, and was unrelated to an independent self-construal. Although not perfectly consistent across issues, these results nonetheless suggest that there is a connection between the identity function of an attitude and cultural mindset in the expected directions.

Other results indicated that stronger endorsement of the individualizing moral foundations was associated with stronger moral conviction across all three issues, but the strength of this relationship varied across issues (with correlations ranging from .13 to .27). Stronger moral conviction was also related to greater endorsement of an independent self-construal for one issue. However, endorsement of the binding moral foundations and interdependent self-construal was unassociated with moral conviction across all three issues.

Finally, the strength of perceiving an attitude as reflective of a personal or social identity function was unrelated to socially desirable responding. Social desirability was positively related to endorsing the binding moral foundations and an independent self-construal and was unrelated to endorsement of the individualizing moral foundations and an interdependent self-construal. Because social desirability was unrelated to or shared less than 2% of the variance with our measures of independent mindset and was also related to one measure of interdependent mindset, these results were inconsistent with the idea that the results of Study 1 might reflect cultural differences in self-views about the social desirability of endorsing a personal identity function relative to a social identity function of an attitude.

### Testing the replicability of Study 1

The second goal of Study 2 was to test the replicability of the pattern of results observed in Study 1. Replicating the approach taken in Study 1, we entered personal and social identity functions in step 1 of the hierarchical regression analysis, and then the binding and individualizing foundations and all two-way interactions between predictors in step 2 separately for all three issues. The three-way interactions were entered in step 3. As can be seen in Table 6, the personal identity function was uniquely positively associated, whereas the social identity function was unassociated, with moral conviction across all three issues—a pattern of results that replicated Study 1. No interactions explained more than 1% of the variance and were therefore interpreted as not meaningful, even if significant.

**Table 6:** Hierarchical regression results (standardized coefficients) for predicting moral conviction across issues in Study 2.

	*Same-sex marriage*				*Gun control*				*Capital punishment*			
Predictor	*β*	*SE β*	*LL*	*UL*	*β*	*SE β*	*LL*	*UL*	*β*	*SE β*	*LL*	*UL*
Step 1												
Personal identity	.47**	.05	.37	.58	.63**	.05	.53	.73	.55**	.05	.44	.65
Social identity	.07	.05	−.03	.18	.04	.05	−.06	.14	<−.01	.05	−.11	.10
*R* ^*2*^_*adj*_	.26				.42				.29			
Step 2												
Individualizing	.18**	.05	.08	.28	.16**	.04	.08	.25	.12*	.05	.03	.21
Binding	−.08	.05	−.18	.02	−.10*	.04	−.19	−.01	−.12*	.05	−.22	−.03
PI x SI	−.04	.05	−.14	.06	−.14**	.04	−.22	−.06	−.07	.05	−.16	.03
PI x Individualizing	.01	.06	−.11	.12	−.08	.05	−.18	.03	.02	.06	−.09	.13
PI x Binding	−.03	.05	−.13	.07	.16**	.05	.06	.26	−.03	.05	−.14	.08
SI x Individualizing	−.07	.06	−.19	.04	.08	.05	−.03	.19	.02	.05	−.09	.12
SI x Binding	<−.01	.05	−.11	.11	−.13*	.05	−.22	−.03	<.01	.05	−.10	.11
*R* ^*2*^_*adj*_	.29				.45				.31			
*ΔR* ^*2*^_*adj*_	.03				.03				.02			
Step 3												
PI x SI x individualizing	.02	.04	−.06	.11	−.03	.05	−.13	.06	−.04	.05	−.13	.06
PI x SI x binding	.08	.04	<−.01	.17	.02	.03	−.04	.09	.07	.04	−.01	.15
*R* ^*2*^_*adj*_	.29				.45				.31			
*ΔR* ^*2*^_*adj*_	.00				.00				.00			

*Note*. *N *= 364 (using complete pairwise observations). PI = personal identity and SI = social identity. LL = lower limit for 95% CI for β; UL = upper limit for 95% CI for β. The significant interactions for the issue of gun control each explained only 1% or less of the variance in the model.

*p < .05. **p < .01.

In summary, the results of Study 2 replicated Study 1. The results were most consistent with the *personal identity hypothesis* and yielded no support for the *social identity* or *combined identity* hypotheses.

### Testing possible boundary conditions: The mindset hypothesis

Another goal of Study 2 was to test whether cultural mindsets moderate the relationship between the identity functions and moral conviction (see Table 6 for the results using the individualizing and binding moral foundations as moderators and S7 Table in S1 File for the results using independent and interdependent self-construal as moderators). As shown in Table 6, only two interactions out of 12 tests for cultural mindset moderation were significant; neither accounted for more than 1% of explained variance in moral conviction. Similarly, non-significant and non-meaningful results emerged for self-construal (see S7 Table in S1 File). Therefore, contrary to the *mindset hypothesis*, cultural mindset (regardless of operationalization) did not consistently or meaningfully moderate the relationship between personal or social identity functions and moral conviction across issues.

### Robustness checks

To investigate how robust support for the *personal identity hypothesis* was in Study 2, we tested whether the observed findings were robust to controls for socially desirable responding, attitude strength, and issue position. The personal identity function was a stronger predictor than the social identity function of moral conviction even when controlling for social desirability, attitude strength (i.e., importance and certainty), and issue position (see S8 Table in S1 File). We also looked at the robustness of the observed findings when applying data exclusion rules (see S9 Table in S1 File), with the pre-registered exclusion rules being that we would exclude participants who took less than 9 minutes to complete the survey (*N* = 74) and who failed one of two open-ended attention checks (*N* = 40). Analyses with and without these exclusions yielded similar conclusions; we reported the analyses without these exclusions to retain the pre-registered target sample size in Table 6. The results of the additional analyses described here were consistent with those in Table 6. In summary, support for the *personal identity hypothesis* was robust even when considering various controls.

## Discussion

The results of Study 2 supported the *personal identity hypothesis* across three issues. Replicating Study 1, attitudes higher in moral conviction were also perceived as more strongly related to people’s sense of personal identity, and not their sense of social identity. We also found that differences in social desirability did not provide a plausible alternative explanation for the observed results. Reporting that attitudes were reflections of personal or social identity functions was virtually uncorrelated with socially desirable responding; not surprisingly, then, controlling for socially desirable responding did not qualify any of our findings.

Surprisingly, we also found no support for the *mindset hypothesis,* that is, the idea that the relationship between personal and social identity functions and moral conviction would depend on whether perceivers’ morality was based more in individualizing or binding moral foundations, or whether perceivers were high versus low in independent or interdependent self-construal. Regardless of cultural mindset, stronger moral conviction about a given issue was associated with stronger perceptions that morally convicted attitudes reflected one’s personal identity.

One strength of our sample for Study 2 was the rich within-culture heterogeneity of cultural mindsets. That said, one could nonetheless argue that the deck was stacked against the *mindset hypothesis* given that we tested hypotheses in a dominantly individualistic cultural context. We addressed this potential limitation in Study 3.

## Study 3

The goal of Study 3 was to examine the relationship between moral conviction and identity functions using two different cultures that vary in their relative degree of individualism and collectivism. The U.S. is one of the most individualistic cultures in the world [101], so personal identity may be a more dominant consideration (or more central to Americans) than it is elsewhere, which may be why we found that moral conviction has stronger connections to personal than social identity as an attitude function in Studies 1 and 2. We might find the reverse pattern of results in less individualistic and more collectivistic cultures. Study 3 therefore tested the generalizability of our conclusions in a more collectivistic culture, specifically India.

We selected India as the comparison point to the U.S. because it tends to have higher collectivism and lower individualism scores than the U.S. On an index for individualism, where 100 is the highest possible score, India ranks at 48 compared to the U.S. ranking of 91 [101]. A meta-analysis found that Americans reported significantly lower collectivism and higher individualism than Indian participants, with a medium effect size for the difference in collectivism and a small effect size for individualism [102].

There are also cultural differences in relevance and understanding of morality that emerge between American and Indian participants, which further bolsters the use of India as the comparison country in the current study. U.S. participants rate harm and fairness foundations as more morally relevant than Indian participants, and Indian participants rate the foundations of ingroup loyalty, authority, and purity as more morally relevant than U.S. participants [103], a finding consistent with the idea that morality may be a more individualistic concept in the U.S. (and therefore will be more strongly related to personal than social identity), and that morality may be a more collectivistic concept in India (and therefore will be more strongly related to social than personal identity).

Prior to running Study 3, we conducted pilot tests with both U.S. and Indian samples to identify issues that were similarly high (or low) in moral conviction for both countries (see S1 Appendix in S1 File in the supplementary materials; participants from both countries were recruited for the pilot study on December 28, 2020). After pilot testing six issues, we selected three issues to use in Study 3 by examining descriptives and reliability for the moral conviction items in each country for equivalence. Thus, in addition to examining cross-cultural differences, we used three different issues from those used in Studies 1 and 2, which allowed us to further test the generalizability of support for the *personal identity hypothesis*.

## Method

### Participants

An *a priori* power analysis was conducted to determine the number of participants needed. Though the previous two studies indicated large effect sizes for the relationship between identity functions and moral conviction, it was unclear what sort of effect size might be expected when examining differences across cultures, so a small effect size estimate was used to be safe (between *R*^2^ of .05 and .1). *R*^*2*^ was converted to *f*^*2*^ for the power analysis. We used the *pwr* package in R. The other parameters entered were 10 predictors used in the final step of the regression analysis (so main effects *and* interaction terms), 80% power, and an alpha of .05. For an *R*^2^ of 0.05, 327 total participants were needed to detect this effect (~ 164 per country) and for an *R*^2^ of 0.1, 150 total participants were needed (75 per country). Participants were recruited to participate in the main study from February 3, 2021 to February 4, 2021 from a pool of participants who completed and passed an English comprehension screener on CloudResearch (for more details, see S2 Appendix in S1 File in supplemental materials; participants were recruited for the screener from January 5, 2021 to February 1, 2021). Our total combined sample size after data cleaning was *N *= 300 (150 American and 150 Indian CloudResearch participants). Data cleaning involved removing preview/test responses (*N *= 2 for the Indian sample and *N *= 2 for American sample) and removing duplicate IDs (including blanks where participants had incomplete responses; *N *= 7 for Indian sample and *N *= 1 for American sample). We also removed missing responses on key main analysis variables (*N *= 14) in the combined data set, so we had a final sample size of *N* = 300 total. Participants ranged in age from 21–83 years old (*M* = 36.44, *SD* = 10.89). 60.13% were male and 39.53% were female. 78.74% indicated the highest level of education they received was a bachelor’s degree or beyond.

## Measures

### Issue position

The same questions were used to measure issue position as in Study 1 except the issues changed. The specific wording for the three issues was the following: “higher taxes on fossil fuels in an effort to reduce greenhouse gases and climate change,” “lifting restrictions on future oil exploration and drilling,” and “banning the use of plastic bags, straws, and cutlery to help protect the environment.”

### Attitude strength

The same measures of moral conviction (α = .86−.89), attitude importance (α = .87−.90), and attitude certainty (α = .91−.92) were used as in the previous two studies.

### Identity function scale

The same subscales from Study 2 were used, with 10 items for the personal identity function subscale (α = .97−.98) and 9 items for the social identity function subscale (α = .97 for all three issues).

### Demographics

The same measures for education, age, and gender from Study 2 were used.

### Attention checks

The attention check items were included as items in the personal identity function subscale for the issue of fossil fuels and the social identity function subscale for the issue of oil exploration. The items were “If you read this question, please select slightly reflected” and “If you read this question, please select ‘not at all reflected”, respectively.

### Procedure

Participants completed a shortened version of the survey used in Study 1 and in Time 2 of Study 2 (with a similar procedure, but only the measures described above). The survey was distributed on CloudResearch separately for each country. It included measures of issue position, attitude strength (including moral conviction), and the identity function scale for each issue. After completing these measures, participants completed demographic measures of age, gender, and education.

## Results

### Confirmatory factor analyses

To confirm that the two-factor model for the identity function scale worked for the Indian and American samples, we conducted confirmatory factor analyses. The results indicated that the two-factor model indeed did work better than the one-factor model (see S10-S11 Tables in S1 File for fit indices and S12-S13 Tables in S1 File for factor loadings), once again confirming that there was a personal identity function factor and social identity function factor.

### Descriptive statistics

Tables 7–9 show the descriptive statistics for the combined sample (see S14-S16 Tables in S1 File for descriptives including covariates with the combined sample and S17-S22 Tables in S1 File for descriptives per country sample). Consistent with the idea that the Indian sample would be higher in collectivism and therefore in the strength of the social identity function of their attitudes, the social identity function measure had higher ratings in the Indian than the American sample (the r values for country and social identity function ranged from .56 to .63 across issues). The idea that the U.S. sample would be higher individualism and therefore in the strength of the personal identity function of their attitudes, however, was not supported. Surprisingly, the personal identity function measure was also higher in the Indian than the American sample (r’s ranged from .20 to .40 across issues), although the strength of these associations was weaker than social identity. The personal and social identity function measures were positively correlated (r’s .54−.55 across issues), such that greater perceptions of a given function were associated with greater moral conviction. Finally, strength of personal identity function was more strongly correlated with moral conviction than social identity function across all three issues (r’s for PI ranged from .78 to .82, and for SI ranged from .44−.49), such that the more people perceived an attitude as reflective of personal identity, the more they also perceived that same attitude as being a moral conviction.

**Table 7:** Descriptive statistics and correlations for issue of fossil fuels.

*Variable*	*M*	*SD*	1	2	3
1. Moral conviction	3.44	1.18			
2. Personal identity	3.46	1.09	.78**		
3. Social identity	2.57	1.22	.47**	.55**	
4. Country			.20**	.26**	.57**

*Note*. *N* = 300 (complete pairwise observations). Country (U.S. = 0, India = 1).

*p < .05. **p < .01.

**Table 8:** Descriptive statistics and correlations for issue of oil exploration.

*Variable*	*M*	*SD*	1	2	3
1. Moral conviction	3.24	1.26			
2. Personal identity	3.33	1.19	.80**		
3. Social identity	2.52	1.21	.44**	.54**	
4. Country			.12*	.20**	.56**

*Note*. *N* = 300 (complete pairwise observations). Country (U.S. = 0, India = 1).

*p < .05. **p < .01.

**Table 9:** Descriptive statistics and correlations for issue of plastic ban.

*Variable*	*M*	*SD*	1	2	3
1. Moral conviction	3.59	1.23			
2. Personal identity	3.61	1.10	.82**		
3. Social identity	2.68	1.31	.49**	.55**	
4. Country			.38**	.40**	.63**

*Note*. *N* = 300 (complete pairwise observations). Country (U.S. = 0, India = 1).

*p < .05. **p < .01.

### Hypothesis tests

Separate hierarchical regression models per issue were conducted to test the *personal identity* and *mindset hypotheses*. The results of the hierarchical regression models are shown in Table 10 (see S23 Table in S1 File for analyses controlling for covariates and S24 Table in S1 File for analyses with data exclusions). Across all three issues, a personal identity function was a unique positive predictor of moral conviction, even when controlling for country. Additionally, the country where participants resided did not meaningfully moderate (i.e., explain more than 1% increase in variance) any effects of personal or social identity functions or their interaction. In summary, regardless of country or issue, the personal identity function was a better predictor of moral conviction than the social identity function, a finding that supports the *personal identity hypothesis* and replicates the results found in Studies 1 and 2.

**Table 10:** Hierarchical regression results (standardized coefficients) for predicting moral conviction across issues in Study 3.

	*Fossil Fuels*				*Oil Exploration*				*Plastic Ban*			
Predictor	*β*	*SE β*	*LL*	*UL*	*β*	*SE β*	*LL*	*UL*	*β*	*SE β*	*LL*	*UL*
Step 1												
Personal identity	.74**	.04	.66	.83	.79**	.04	.71	.87	.78**	.04	.70	.86
Social identity	.06	.04	−.02	.15	.02	.04	−.06	.10	.06	.04	−.02	.14
*R* ^*2*^_*adj*_	.61				.64				.67			
Step 2												
Country	−.09	.09	−.27	.08	−.17*	.08	−.34	−.01	.11	.09	−.06	.28
PI*SI	.01	.05	−.08	.11	−.03	.05	−.13	.07	.05	.05	−.05	.15
PI x Country	−.12	.10	−.33	.09	−.25*	.10	−.44	−.06	−.28*	.10	−.48	−.09
SI x Country	−.02	.11	−.24	.20	.10	.10	−.10	.30	.04	.10	−.15	.23
*R* ^*2*^_*adj*_	.61				.65				.67			
*ΔR* ^*2*^	.00				.01				.00			
Step 3												
PI x SI x Country	.02	.10	−.17	.22	−.13	.10	−.33	.07	−.13	.11	−.34	.09
*R* ^*2*^_*adj*_	.60				.65				.67			
*ΔR* ^*2*^	−.01				.00				.00			

*Note*. *N *= 300. PI = personal identity and SI = social identity. LL = lower limit for 95% CI for β; UL = upper limit for 95% CI for β. The significant interactions for the issues of oil exploration and plastic ban did not explain more than 1% of the variance in the model.

*p < .05. **p < .01.

## Discussion

Like Studies 1 and 2, the results of Study 3 provided robust support for the *personal identity hypothesis.* A personal identity function emerged as the strongest predictor of moral conviction, regardless of country. Once the strength of a personal identity function was accounted for, a social identity function did not predict moral conviction directly or contingently as a function of country, a result at odds with the *mindset hypothesis*.

## General Discussion

We found consistent support for the *personal identity hypothesis* across six issues and three studies. Attitudes that people perceived they held for personal identity reasons were also uniquely perceived as stronger moral convictions. We did not find support for the *social identity hypothesis* or *combined identity hypothesis*, because perceiving attitudes as reflective of social identity concerns was not a unique predictor of stronger moral convictions nor was there an interactive effect of personal and social identity concerns on moral conviction. This pattern of results also held even when controlling for attitude importance, attitude certainty, and social desirability and when testing for the possible moderating roles of cultural mindsets. This finding is consistent with other research that has found that the personal self has more primacy/sovereignty over relational and collective (group) selves (e.g., [104,105]). For example, even when a fused ingroup identity was made salient, people rated their personal selves as more valuable, felt a greater impact if they imagined losing their personal identity, and reported feeling that their personal selves were more representative of the “real you” than their collective selves [104]. Similarly, people have a stronger desire to protect and enhance their individual selves than their relational or collective selves (e.g., [105]).

Our results are also consistent with an explanation for what has been called the “moral mandate effect,” that is, that people are more concerned about achieving moralized ends than the procedures used to achieve them (e.g., [4]). Although people more readily accept *unfavorable* outcomes when they are the result of fair and unbiased procedures that allow for voice, largely because fair procedures reinforce people’s sense of belongingness in the group (i.e., their social identity needs), this effect does not emerge when people have a *moral* investment in the outcomes at hand, that is, when they have a moral conviction about the outcome (e.g., that abortion should be legal or illegal). Researchers have theorized that this effect results from people’s personal identity concerns looming larger than their social identity concerns when they have strong moral convictions about outcomes (e.g., [43]). Because they are more concerned with expressing their sense of moral authenticity or conscience in these cases, they are less concerned about group belongingness, standing, and respect and, therefore, are less attentive to the procedures used to decide the outcome than they are that the “correct” outcome is achieved. Our findings are consistent with this interpretation of the moral mandate effect by more directly testing and establishing that there is a stronger association of moral conviction with a personal than social identity function.

The results suggest that people may hold moral convictions for more personal identity reasons, which is important for several reasons. One reason is that it could provide insight into why experiencing a violation of one’s worldview leads to such outrage and discomfort – it might represent a threat to and loss of one’s personal identity. Another reason why the results matter is that they suggest that increasing motivations core to one’s personal identity or crafting persuasive messages that appeal to one’s personal identity may encourage people to become more politically engaged via behaviors such as voting or activism.

One strength of the approach we took with the current studies is that we explicitly tested the relationship between perceptions of the personal identity versus social identity functions and attitudes held with moral conviction, rather than relying on indirect support for the relationship between personal and/or social identity and moral conviction (e.g., [4]). Furthermore, to our knowledge, this is the first study that has included measures that allow for direct comparisons of the relationship between personal versus social identity functions and moral conviction (previous studies measured or allowed for inferences about one or the other, but not both in the same study, e.g., [5]). Besides including personal and social identity function measures in each study, we also had built-in replications across six issues and multiple samples. Finding consistent patterns of results with multiple replications provides greater confidence in the conclusion that morally convicted attitudes serve more of a personal identity than social identity expressive function.

Because morality has an important role in group-based dynamics and motivations (e.g., [55]), some believe that moral conviction should have a strong social group component. To the extent that different cultural backgrounds emphasize greater or less importance of others when thinking of one’s self-concept, this would presumably be reflected in one’s moral convictions. However, we found that the personal identity function was the only unique predictor of moral conviction, regardless of the perceiver’s cultural orientation or context. Our findings are, therefore, more consistent with theories that posit that people’s moral convictions are more closely connected to their conceptions of themselves as being personally morally authentic and agents of personal conscience than they are to concerns about their positions as members of groups or their interpersonal and group relationships (e.g., [37]).

The finding that a personal identity function is a better predictor of moral conviction than a social identity function may seem at odds with some previous research that found a relationship between social identity and moral conviction. For example, the Social Identity Model of Collective Action (SIMCA) highlights a relationship between politicized (social) identities and moral conviction as important for motivating collective action tendencies and behavior. Studies testing SIMCA hypotheses find that stronger moral convictions about a given cause are associated with heightened politicized identification with the issue-oriented group, that in turn is associated with an increased likelihood of engaging in cause-related collective action (e.g., [5,7]).

However, these studies did not include a measure of personal identity (e.g., personal identity function) as a point of comparison and the measure of politicized identification is more of a proxy than direct measure of a social identity function. One way to reconcile these findings with the current work is that even though people’s moral convictions may be held more to express certain aspects of personal identity (e.g., personal conscience) than social identity, people acting in the name of personal conscience may also be particularly motivated to find like-minded others to help advance their cause, which in turn could then lead them to also develop a related group identity. To further understand the relative roles of personal versus social identity in predicting intentions to engage in collective action, we need studies that include measures of both variables and tests of their comparative predictive power (see [106] on the value of negative in addition to positive testing strategies to allow for stronger inferences), and study designs that can test hypotheses about causal order.

One could argue that one reason we didn’t observe stronger associations of a social identity function and moral conviction, or moderating effects of cultural worldview, is because we relied on self-reports of these variables, which capture people’s perceptions of their attitudes and how they relate to moral conviction. People may not always have sufficient ability, knowledge, or insight to report accurately and reliably why they believe something or act in a certain manner (e.g., [107–109]). Applied to the current research, the internalization of people’s social identities may be so complete that people are unaware of how much a given attitude is influenced by their group membership (e.g., see [110,111] for examples). This argument, however, would require that people have a greater self-awareness of the degree to which a given attitude is influenced by personal conscience or concerns about moral authenticity than an awareness of the degree to which an attitude reflects their concerns about belongingness, reputation, or being a good group member. Given the salience of belongingness as a fundamental human need or motivation [112], the idea that people are more consciously aware of their personal than their social identity concerns and needs is an unlikely alternative explanation for the findings observed here.

One could also argue that people’s personal identities include their social identities—in other words, social identities can become so internalized they become incorporated into people’s sense of personal self, and therefore perceptions of personal versus social identity concerns could become muddled. This perspective on the (in)distinctiveness of personal and social identity would potentially make social identity theory become tautological and untestable, where anything that one might attribute to personal identity would be assumed to be due to social identity; or if personal identity explains something more than social identity, it is only because the former contains the latter. In other words, if group memberships and their associated needs and influences on one’s identity and self-concept are part of personal identity, then it makes it difficult to understand how to test the influences of different psychological needs related to different types of identity if these aspects of the self are in fact inseparable.

The level of distinction that can or should be made between personal identity and social identities can vary, however, depending on the theoretical approach taken and the understanding of that theoretical approach (e.g., comparing levels/aspects of identity from a social identity theory perspective versus a cultural perspective). Some theorists, for example, argue that the self-concept is social by nature, but this does not mean that everything that is social identity is personal identity or vice versa. Rather, when people develop their self-concept (that includes both personal and social identities), key social contexts and situational factors influence their formation. People can develop a sense of self that reflects group memberships, as well as a sense of self that involves unique characteristics not associated with the group, and then depending on the situation at hand, they may be motivated to think and act more from their personal identity or one or more key social identities (e.g., [113]).

A critique from this perspective on social identity theory may be that asking people about personal identity without indicating “irrespective of the group” could make people think of the self in terms of both their personal and social identities. However, we were interested in the psychological needs stemming from personal versus social identity in the current study, not the identity content itself. The current measures of identity functions can be answered in a way that appropriately captures distinct personal identity versus social identity needs, even in the absence of specific direction for participants to think about the personal identity items without taking group concerns into account. Specifically, needs like authenticity and consistency are more likely to stem from personal identity (the “core, enduring” part of the self that allows people to see themselves as the same person over time and context), whereas social identity needs like belonging are reflected in people’s cognitive and emotional ties to key social groups. These psychological needs are what we capture in the current identity function measures.

In addition, even though data based on self-reports have known limitations, self-reports are nonetheless considered informative for several reasons. The idea that people are the best-qualified witnesses to their own motives is supported by the “indisputable fact that no one else has access to more information,” including the opportunity to observe themselves over vast periods of time and contexts [114]. Moreover, the self has access to intrapsychic information—thoughts, feelings, and motivations—that are unavailable to others [115]. People’s beliefs about themselves have a powerful influence on their thoughts, feelings, and behavior, and are, therefore, worthy of study. For example, although believing oneself to be shy may not be the same as being genetically shy, the belief can nonetheless have a powerful influence on one’s behavior [116]. Similarly, believing that one’s attitudes are based on concerns about moral authenticity and consistency—and not based on concerns about reputation or being a good group member—is likely to have a number of implications. For example, these beliefs may contribute to why people are less likely to conform to majority group opinion or to obey legitimate authorities whose dictates are at odds with their personal beliefs when those beliefs are held with strong moral conviction (e.g., [17,117]. Other research similarly finds that self-perceptions affect a host of meaningful outcomes, including behavior [116,118,119], how people present themselves to others [120], and who they choose to affiliate with [121].

Future research investigating the role of identity functions and moral conviction would benefit from taking a more longitudinal or developmental approach, which could find that the intersections of social and personal identity and moral conviction are more complex than we can capture with the cross-sectional approach taken here. For example, moral convictions may be based more on social than personal identity considerations in development. For example, moralized positions on various issues may emerge because people internalize the injunctive norms of the groups they interact and identify with during childhood and adolescence. These, in turn, could shape people’s sense of themselves as morally authentic and serve as the foundation for developing a strong sense of personal identity. Over time, people may come to interpret that their attitudes are more closely tied to their sense of personal identity (a more proximal association), when they were more crucially shaped by group norms and group commitments developmentally (a more distal association).

We also do not yet know the causal direction of the relationship between the identity functions of attitudes and moral conviction (i.e., what comes first—a meta-cognition that an attitude is identity-relevant or a meta-cognition that an attitude reflects a moral conviction?), since the current studies employed correlational designs. Future research should experimentally study the connections between identity functions and moral conviction, for example, by manipulating the salience of a personal identity function or social identity function of specific attitudes and measuring moral conviction as the outcome (or vice versa).

Additionally, it is possible that we found weaker connections between a social identity function and moral conviction because we asked about the former without specifying a group. Alternatively, one could argue that the issues we selected to study might pull more for personal than social identity expression. Although not out of the question, replicating the finding of a stronger association between a personal versus social identity function and moral conviction across six different issues and two cultural settings shifts the burden of proof to one who would argue that issue selection is a limiting factor.

Finally, it is important to emphasize a few key caveats to the conclusion that moral convictions serve a personal identity function. First, even though the personal identity function was more strongly related to moral conviction than the social identity function, a) the observed effect sizes are small (unique explained variance ranged from 1% to 8% across issues/samples; see S25-S27 Tables in S1 File), and b) the personal identity function was consistently either a similar or weaker predictor of moral conviction than perceived attitude importance and certainty (see S3, S8, S23, S25-S27 Tables in S1 File). Taken together, although moral convictions may serve a personal identity function, they may serve other unmeasured functions as well.

Second, because we specifically tested the identity functions of morally convicted attitudes, our results may only apply to our understanding of moral conviction as a phenomenon. The word “moral” is attached to many phenomena in psychology, such as judgments of responsibility and blameworthiness, reasoning about moral-based decisions, moral licensing, behaving in a moral or ethical way, and understanding the extent to which people have traits related to morality, just to name a few (e.g., [29]). Some of these other aspects of morality may be more strongly influenced by social than personal identity concerns. For example, unlike moral convictions, judgments of blame and responsibility seem likely to be based more on group norms and social learning than people’s personal conceptions of morality (e.g., [30]).

## Conclusion

The current studies expand our understanding of moral conviction and identity by establishing that a personal identity function is a reliable predictor of moral conviction, a result that was consistent across multiple issues, samples, and controlling for a variety of covariates. Moreover, the association between a personal identity function and moral conviction is robust across individual or cultural measures of cultural mindset. These results suggest that moral convictions are more about people’s sense of themselves as morally and personally authentic than about people’s attachments to specific groups or group norms.

## Supporting information

S1 File File provides supplemental tables (S1-S27 Tables) with results of additional analyses done and includes details about the pilot study (S1 Appendix) and screener study (S2 Appendix) done as part of Study 3 in the main manuscript. (DOCX)
