# Supplementary material for: Understanding the functional basis of moral conviction: Is moral conviction related to personal and social identity expression?
Source: PLoS One. 2025 Jul 3;20(7):e0327438. doi: 10.1371/journal.pone.0327438 (PMC12225793; doi:10.1371/journal.pone.0327438)
Supplement: S1 File — (DOCX) [file pone.0327438.s001.docx]

**S1 File**

**Supporting Information Materials**

The following supplemental tables (S1-S27 Tables) provide results of additional analyses done (e.g., factor analyses, robustness checks, descriptive analyses for different samples). S1 and S2 Appendices provide details about a pilot study and screener study done as part of Study 3 in the main manuscript.

**S1 Table. Factor Loadings > .3 from Exploratory Factor Analyses of the Personal and Social Identity Function Scale Items for the issues of Same-sex Marriage, Gun Control, and Capital Punishment using Oblimin Rotation in Study 1**

|  | *Same-sex marriage* |  |  | *Gun control* |  |  | *Capital punishment* |  |
| --- | --- | --- | --- | --- | --- | --- | --- | --- |
|  | Factor 1: Personal  Identity | Factor 2: Social identity |  | Factor 1: Personal  Identity | Factor 2: Social identity |  | Factor 1: Personal  Identity | Factor 2: Social identity |
| Eigenvalue | 12.44 | 2.73 |  | 12.80 | 2.76 |  | 12.93 | 2.95 |
| % Variance | 36.80 | 28.70 |  | 37.50 | 30.00 |  | 35.90 | 34.40 |
| My sense of who I am as a person | .91 |  |  | .89 |  |  | .93 |  |
| My true self | .98 |  |  | .95 |  |  | .96 |  |
| The real me | .99 |  |  | .97 |  |  | .97 |  |
| My core self | .98 |  |  | .98 |  |  | .95 |  |
| Who I am as a person | .92 |  |  | .97 |  |  | .96 |  |
| My own personal well-being and self-esteem | .73 |  |  | .75 |  |  | .68 |  |
| Important part of who I am | .77 |  |  | .79 |  |  | .72 |  |
| My ideas about what kind of person I really am | .86 |  |  | .84 |  |  | .85 |  |
| My private opinions of myself | .65 |  |  | .68 |  |  | .69 |  |
| My internal guiding principles | .69 |  |  | .77 |  |  | .79 |  |
| How I feel about important others in my life | .46 | .37 |  | .34 | .42 |  |  | .61 |
| My desire to maintain close relationships |  | .74 |  |  | .75 |  |  | .88 |
| My desire to avoid unnecessary conflict with others |  | .82 |  |  | .63 |  |  | .85 |
| A signal to others that I am a good group member |  | .72 |  |  | .84 |  |  | .82 |
| Values of the group of people most important to me |  | .64 |  |  | .78 |  |  | .75 |
| My identification with central groups in my life |  | .67 |  |  | .78 |  |  | .81 |
| My desire to avoid being rejected by others who are important to me |  | .90 |  |  | .95 |  |  | .90 |
| Reputation (what others think of me) |  | .78 |  |  | .88 |  |  | .83 |
| Feelings of connectedness with those who I am close to |  | .85 |  |  | .90 |  |  | .88 |
| Relationships with those I feel close to |  | .84 |  |  | .75 |  |  | .94 |
| External factors that guide my principles and values |  | .62 |  | .38 | .39 |  | .32 | .45 |

*Note*. *N* = 322 (using complete pairwise observations).

**S2 Table. Factor Loadings > .3 from Exploratory Factor Analyses of the Personal and Social Identity Function Scale Items for Issues of Same-sex Marriage, Gun Control, and Capital Punishment using Varimax Rotation in Study 1**

|  | *Same-sex marriage* |  |  | *Gun control* |  |  | *Capital punishment* |  |
| --- | --- | --- | --- | --- | --- | --- | --- | --- |
|  | Factor 1: Personal  Identity | Factor 2: Social identity |  | Factor 1: Personal  Identity | Factor 2: Social identity |  | Factor 1: Personal  Identity | Factor 2: Social identity |
| Eigenvalue | 12.44 | 2.73 |  | 12.80 | 2.76 |  | 12.93 | 2.95 |
| % Variance | 38.70 | 30.80 |  | 38.70 | 33.00 |  | 37.10 | 36.20 |
| My sense of who I am as a person | .88 |  |  | .85 | .32 |  | .87 |  |
| My true self | .92 |  |  | .89 |  |  | .90 |  |
| The real me | .92 |  |  | .91 |  |  | .90 |  |
| My core self | .92 |  |  | .91 |  |  | .89 |  |
| Who I am as a person | .87 |  |  | .90 |  |  | .89 |  |
| My own personal well-being and self-esteem | .74 | .34 |  | .73 |  |  | .71 | .40 |
| Important part of who I am | .78 | .36 |  | .79 | .37 |  | .75 | .43 |
| My ideas about what kind of person I really am | .83 | .30 |  | .83 | .36 |  | .81 |  |
| My private opinions of myself | .66 | .33 |  | .71 | .40 |  | .71 | .38 |
| My internal guiding principles | .70 | .31 |  | .74 |  |  | .74 |  |
| How I feel about important others in my life | .56 | .49 |  | .45 | .51 |  | .40 | .65 |
| My desire to maintain close relationships | .38 | .74 |  | .38 | .77 |  |  | .82 |
| My desire to avoid unnecessary conflict with others | .20 | .75 |  |  | .62 |  |  | .80 |
| A signal to others that I am a good group member | .33 | .71 |  | .30 | .81 |  | .31 | .80 |
| Values of the group of people most important to me | .40 | .67 |  | .36 | .78 |  | .37 | .76 |
| My identification with central groups in my life | .41 | .69 |  | .37 | .78 |  | .31 | .79 |
| My desire to avoid being rejected by others who are important to me |  | .80 |  |  | .85 |  |  | .83 |
| Reputation (what others think of me) |  | .70 |  |  | .79 |  |  | .78 |
| Feelings of connectedness with those who I am close to | .31 | .81 |  |  | .85 |  |  | .84 |
| Relationships with those I feel close to | .34 | .81 |  | .36 | .76 |  |  | .88 |
| External factors that guide my principles and values | .37 | .63 |  | .48 | .49 |  | .44 | .53 |

*Note*. *N* = 322 (using complete pairwise observations).

**S3 Table. Hierarchical Regression Results (Standardized Coefficients) for Predicting Moral Conviction Across Issues Controlling for Covariates in Study 1**

|  | *Same-sex marriage* |  |  |  | *Gun control* |  |  |  | *Capital punishment* |  |  |  |  |
| --- | --- | --- | --- | --- | --- | --- | --- | --- | --- | --- | --- | --- | --- |
| Predictor | *β* | *SE β* | *LL* | *UL* | *β* | *SE β* | *LL* | *UL* | *β* | *SE β* | *LL* | *UL* |  |
| Step 1 |  |  |  |  |  |  |  |  |  |  |  |  |  |
| Attitude importance | .35** | .04 | .26 | .43 | .57** | .05 | .47 | .67 | .47** | .05 | .37 | .58 |  |
| Attitude certainty | .60** | .05 | .51 | .69 | .27** | .05 | .16 | .37 | .35** | .05 | .24 | .45 |  |
| Issue position | -.17** | .04 | -.24 | -.10 | <-.01 | .04 | -.07 | .07 | -.01 | .04 | -.08 | .06 |  |
| *R^2^_adj_* | .67 |  |  |  | .63 |  |  |  | .59 |  |  |  |  |
| Step 2 |  |  |  |  |  |  |  |  |  |  |  |  |  |
| Personal identity | .18** | .05 | .08 | .28 | .18** | .05 | .07 | .28 | .27** | .05 | .17 | .37 |  |
| Social identity | -.03 | .04 | -.11 | .05 | -.01 | .04 | -.10 | .07 | -.13** | .05 | -.22 | -.04 |  |
| *R^2^_adj_* | .68 |  |  |  | .64 |  |  |  | .62 |  |  |  |  |
| *ΔR^2^_adj_* | | .01 |  |  |  | .01 |  |  |  | .03 |  |  |  |
| Step 3 |  |  |  |  |  |  |  |  |  |  |  |  |  |
| Personal identity*Social identity | .01 | .03 | -.05 | .08 | -.06 | .03 | -.13 | .01 | -.09* | .03 | -.16 | -.02 |  |
| *R^2^_adj_* | .68 |  |  |  | .64 |  |  |  | .63 |  |  |  |  |
| *ΔR^2^_adj_* | .00 |  |  |  | .00 |  |  |  | .01 |  |  |  |  |

*Note*. *N* = 320. LL = lower limit for 95% CI for β; UL = upper limit for 95% CI for β. Compared to results in main text, these analyses show that for the issue of capital punishment, there was a significant but small interaction of personal identity (PI) x social identity (SI) (*R^2^* change of .01, so only accounted for 1% of the variance), and social identity was a significant negative predictor of moral conviction, which did not align with our hypothesis. For the issue of gun control, the PI x SI interaction was no longer significant.

**p* < .05. ***p* < .01

**S4 Table. Hierarchical Regression Analyses (Standardized Coefficients) Predicting Moral Conviction Without Covariates Across Issues in Study 1**

|  | *Same-sex marriage* |  |  |  | *Gun control* |  |  |  | *Capital punishment* |  |  |  |
| --- | --- | --- | --- | --- | --- | --- | --- | --- | --- | --- | --- | --- |
| Predictor | *β* | *SE β* | *LL* | *UL* | *β* | *SE β* | *LL* | *UL* | *β* | SE *β* | LL | UL |
| Step 1 |  |  |  |  |  |  |  |  |  |  |  |  |
| Personal identity | .63** | .06 | .52 | .75 | .68** | .06 | .56 | .80 | .57** | .06 | .45 | .69 |
| Social identity | .02 | .06 | -.10 | .14 | -.01 | .06 | -.14 | .11 | .03 | .06 | -.10 | .16 |
| *R^2^_adj_* | .40 |  |  |  | .43 |  |  |  | .36 |  |  |  |
| Step 2 |  |  |  |  |  |  |  |  |  |  |  |  |
| Personal identity*Social identity | -.06 | .05 | -.16 | .04 | -.08 | .06 | -.20 | .03 | -.04 | .05 | -.14 | .06 |
| *R^2^_adj_* | .40 |  |  |  | .43 |  |  |  | .36 |  |  |  |
| *ΔR^2^_adj_* | .00 |  |  |  | .00 |  |  |  | .00 |  |  |  |

*Note*. Analyses with data exclusion criteria applied (i.e., participants who took less than 5 minutes to complete the study (N = 3) or did not pass at least 2 of the 3 attention checks (N = 59); Final *N* = 258). LL = lower limit for 95% CI for β; UL = upper limit for 95% CI for β.

**p* < .05. ** *p* < .01.

**S5 Table. Goodness-of-Fit Indicators for Confirmatory Factor Analysis Models for Personal and Social Identity Function Items in Study 2**

| Model | X^2^ | df | X^2^ diff | CFI | BIC | RMSEA |
| --- | --- | --- | --- | --- | --- | --- |
|  | SSM |  |  |  |  |  |
| One-factor | 2459.50 | 152 |  | .66 | 20209.86 | .20 |
| Two-factor | 1179.70 | 151 | 1279.80** | .85 | 18935.96 | .14 |
|  | GC |  |  |  |  |  |
| One-factor | 2080.54 | 152 |  | .71 | 18810.34 | .19 |
| Two-factor | 891.72 | 151 | 1188.80** | .89 | 17627.41 | .12 |
|  | CP |  |  |  |  |  |
| One-factor | 2867.48 | 152 |  | .64 | 18410.96 | .22 |
| Two-factor | 923.38 | 151 | 1944.10** | .90 | 16472.76 | .12 |

*Note*. *N* = 367 (using complete pairwise observations). SSM = same-sex marriage; GC = gun control; CP = capital punishment; CFI = comparative fit index; BIC = Bayesian information criterion; RMSEA = root-mean-square error of approximation.

**p* < .05. ***p* < .01.

**S6 Table. Standardized Factor Loadings for 2-factor Confirmatory Model for Identity Function Items for Issues of Same-sex Marriage, Gun control, and Capital Punishment in Study 2**

|  | SSM | GC | CP | SSM | GC | CP |
| --- | --- | --- | --- | --- | --- | --- |
|  | Factor 1: PI |  |  | Factor 2: SI |  |  |
| My sense of who I am as a person | .90 | .91 | .93 |  |  |  |
| My true self | .97 | .96 | .93 |  |  |  |
| The real me | .94 | .95 | .94 |  |  |  |
| My core self | .96 | .93 | .94 |  |  |  |
| Who I am as a person | .90 | .91 | .94 |  |  |  |
| My own personal well-being and self-esteem | .69 | .65 | .70 |  |  |  |
| Important part of who I am | .76 | .82 | .80 |  |  |  |
| My ideas about what kind of person I really am | .77 | .82 | .80 |  |  |  |
| My private opinions of myself | .67 | .71 | .72 |  |  |  |
| My internal guiding principles | .74 | .75 | .77 |  |  |  |
| My desire to maintain close relationships |  |  |  | .81 | .83 | .86 |
| My desire to avoid unnecessary conflict with others |  |  |  | .61 | .72 | .81 |
| A signal to others that I am a good group member |  |  |  | .74 | .80 | .80 |
| Values of the group of people most important to me |  |  |  | .78 | .75 | .81 |
| My identification with central groups in my life |  |  |  | .76 | .80 | .83 |
| My desire to avoid being rejected by others who are important to me |  |  |  | .61 | .70 | .79 |
| Reputation (what others think of me) |  |  |  | .56 | .67 | .76 |
| Feelings of connectedness with those who I am close to |  |  |  | .85 | .79 | .87 |
| Relationships with those I feel close to |  |  |  | .82 | .78 | .86 |

*Note*. *N* = 367 (complete pairwise observations). SSM = same-sex marriage, GC = gun control, CP = capital punishment, PI = personal identity, SI = social identity.

**S7 Table. Hierarchical Regression Results (Standardized Coefficients) for Predicting Moral Conviction Across Issues using Self-Construal as Mindset Operationalization in Study 2**

|  | *Same-sex marriage* |  |  | |  | *Gun control* |  |  |  | *Capital punishment* |  |  |  |
| --- | --- | --- | --- | --- | --- | --- | --- | --- | --- | --- | --- | --- | --- |
| Predictor | *β* | *SE β* | *LL* | *UL* | | *β* | *SE β* | *LL* | *UL* | *β* | *SE β* | *LL* | *UL* |
| Step 1 |  |  |  |  | |  |  |  |  |  |  |  |  |
| Personal identity | .47** | .05 | .37 | .58 | | .63** | .05 | .53 | .73 | .55** | .05 | .44 | .65 |
| Social identity | .07 | .05 | -.03 | .18 | | .04 | .05 | -.06 | .14 | <-.01 | .05 | -.10 | .11 |
| *R^2^_adj_* | .26 |  |  |  | | .42 |  |  |  | .29 |  |  |  |
| Step 2 |  |  |  |  | |  |  |  |  |  |  |  |  |
| Independent SC | .07 | .05 | -.03 | .17 | | .09* | .04 | <.01 | .18 | .06 | .05 | -.04 | .15 |
| Interdependent SC | -.07 | .05 | -.17 | .03 | | .01 | .04 | -.08 | .09 | -.09 | .05 | -.18 | .01 |
| PI*SI | -.07 | .04 | -.15 | .02 | | -.10* | .04 | -.18 | -.03 | -.06 | .04 | -.15 | .02 |
| PI x Independent SC | -.07 | .05 | -.17 | .04 | | .01 | .05 | -.09 | .11 | -.04 | .06 | -.15 | .06 |
| PI x Interdependent SC | -.09 | .05 | -.02 | .19 | | .05 | .05 | -.04 | .14 | .02 | .05 | -.08 | .12 |
| SI x Independent SC | <-.01 | .06 | -.12 | .11 | | .01 | .05 | -.09 | .11 | .06 | .05 | -.04 | .17 |
| SI x Interdependent SC | .02 | .06 | -.10 | .14 | | -.05 | .05 | -.14 | .05 | -.02 | .05 | -.12 | .08 |
| *R^2^_adj_* | .27 |  |  |  | | .43 |  |  |  | .29 |  |  |  |
| *ΔR^2^_adj_* | .01 |  |  |  | | .01 |  |  |  | .00 |  |  |  |
| Step 3 |  |  |  |  | |  |  |  |  |  |  |  |  |
| PI x SI x Independent SC | -.03 | .05 | -.13 | .06 | | -.06 | .04 | -.14 | .02 | -.05 | .04 | -.14 | .04 |
| PI x SI x Interdependent SC | .01 | .04 | -.08 | .10 | | .04 | .04 | -.04 | .11 | .08* | .04 | <.01 | .16 |
| *R^2^_adj_* | .27 |  |  |  | | .43 |  |  |  | .30 |  |  |  |
| *ΔR^2^_adj_* | .00 |  |  |  | | .00 |  |  |  | .01 |  |  |  |

*Note*. *N* = 364 (using complete pairwise observations). LL = lower limit for 95% CI for β; UL = upper limit for 95% CI for β. PI = personal identity, SI = social identity, SC = self-construal. The significant interactions for the issue of gun control and capital punishment explained only 1% or less of the variance in the model.

**p* < .05. ***p* < .01.

**S8 Table. Hierarchical Regression Results (Standardized Coefficients) for Predicting Moral Conviction Across Issues Controlling for All Covariates in Study 2**

|  | *Same-sex marriage* |  |  |  | *Gun control* |  |  |  | *Capital punishment* |  |  |  |
| --- | --- | --- | --- | --- | --- | --- | --- | --- | --- | --- | --- | --- |
| Predictor | *β* | *SE β* | *LL* | *UL* | *β* | *SE β* | *LL* | *UL* | *β* | *SE β* | *LL* | *UL* |
| Step 1 |  |  |  |  |  |  |  |  |  |  |  |  |
| Attitude importance | .27** | .05 | .18 | .36 | .46** | .05 | .37 | .56 | .29** | .05 | .18 | .40 |
| Attitude certainty | .58** | .04 | .49 | .67 | .33** | .05 | .23 | .42 | .39** | .05 | .29 | .50 |
| Issue position | -.16** | .04 | -.24 | -.09 | -.08* | .04 | -.15 | -.01 | -.09 | .04 | -.17 | -.01 |
| Social desirability | .03 | .04 | -.04 | .10 | -.05 | .04 | -.12 | .02 | -.09 | .04 | -.17 | -.01 |
| *R^2^_adj_* | .52 |  |  |  | .54 |  |  |  | .38 |  |  |  |
| Step 2 |  |  |  |  |  |  |  |  |  |  |  |  |
| Personal identity | .17** | .05 | .08 | .27 | .34** | .05 | .25 | .43 | .32** | .06 | .21 | .43 |
| Social identity | -.01 | .05 | -.10 | .08 | -.04 | .04 | -.12 | .04 | -.06 | .05 | -.16 | .03 |
| *R^2^_adj_* | .54 |  |  |  | .60 |  |  |  | .43 |  |  |  |
| *ΔR^2^_adj_* | .02 |  |  |  | .06 |  |  |  | .05 |  |  |  |
| Step 3 |  |  |  |  |  |  |  |  |  |  |  |  |
| Individualizing | .08 | .04 | -.01 | .16 | .12** | .04 | .05 | .19 | .12** | .04 | .04 | .21 |
| Binding | -.01 | .04 | -.10 | .07 | -.06 | .04 | -.13 | .02 | -.12* | .05 | -.21 | -.03 |
| Personal identity*Social identity | -.05 | .04 | -.13 | .03 | -.10* | .04 | -.17 | -.03 | -.06 | .04 | -.14 | .03 |
| PI x Individualizing | .10* | .05 | .01 | .19 | -.02 | .05 | -.11 | .07 | .01 | .05 | -.09 | .10 |
| PI x Binding | -.04 | .04 | -.12 | .05 | .10* | .04 | .02 | .19 | -.01 | .05 | -.11 | .09 |
| SI x Individualizing | -.07 | .05 | -.16 | .03 | .02 | .05 | -.07 | .12 | .01 | .05 | -.09 | .10 |
| SI x Binding | -.02 | .04 | -.11 | .06 | -.08* | .04 | -.17 | <-.01 | <.01 | .05 | -.09 | .10 |
| *R^2^_adj_* | .54 |  |  |  | .62 |  |  |  | .44 |  |  |  |
| *ΔR^2^_adj_* | .00 |  |  |  | .02 |  |  |  | .01 |  |  |  |
| Step 4 |  |  |  |  |  |  |  |  |  |  |  |  |
| PI x SI x individualizing | .02 | .03 | -.05 | .09 | -.05 | .04 | -.13 | .03 | -.04 | .04 | -.13 | .04 |
| PI x SI x binding | .05 | .04 | -.02 | .12 | .04 | .03 | -.01 | .10 | .04 | .04 | -.03 | .11 |
| *R^2^_adj_* | .54 |  |  |  | .62 |  |  |  | .44 |  |  |  |
| *ΔR^2^_adj_* | .00 |  |  |  | .00 |  |  |  | .00 |  |  |  |

*Note*. *N* = 364 (using complete pairwise observations). LL = lower limit for 95% CI for β; UL = upper limit for 95% CI for β. PI = personal identity, SI = social identity. For the issue of gun control, the PI x SI interaction accounted for 1% of variance, and the interactions with the binding foundations accounted for less than 1% of the variance.

**p* < .05. ***p* < .01.

**S9 Table. Hierarchical Regression Results (Standardized Regression Coefficients) for Predicting Moral Conviction Across Issues Without Covariates in Study 2**

|  | *Same-sex marriage* |  |  |  | *Gun control* |  |  |  | *Capital punishment* |  |  |  |
| --- | --- | --- | --- | --- | --- | --- | --- | --- | --- | --- | --- | --- |
| Predictor | *β* | *SE β* | *LL* | *UL* | *β* | *SE β* | *LL* | *UL* | *β* | *SE β* | *LL* | *UL* |
| Step 1 |  |  |  |  |  |  |  |  |  |  |  |  |
| Personal identity | .42** | .06 | .30 | .54 | .62** | .06 | .50 | .74 | .55** | .06 | .42 | .67 |
| Social identity | .08 | .06 | -.05 | .20 | .05 | .06 | -.07 | .18 | <.01 | .06 | -.12 | .13 |
| *R^2^_adj_* | .23 |  |  |  | .42 |  |  |  | .30 |  |  |  |
| Step 2 |  |  |  |  |  |  |  |  |  |  |  |  |
| Individualizing | .09 | .06 | -.03 | .22 | .13* | .06 | -.02 | .24 | .02 | .06 | -.09 | .14 |
| Binding | -.06 | .06 | -.18 | .06 | -.11* | .05 | -.22 | -.01 | -.11 | .06 | -.22 | .01 |
| Personal identity*Social identity | .01 | .06 | -.11 | .13 | -.19** | .05 | -.29 | -.09 | -.10 | .05 | -.20 | .01 |
| PI x Individualizing | .07 | .07 | -.06 | .21 | .03 | .07 | -.11 | .17 | .07 | .07 | -.08 | .22 |
| PI x Binding | .06 | .07 | -.19 | .08 | .15* | .06 | .03 | .26 | -.09 | .06 | -.22 | .04 |
| SI x Individualizing | -.12 | .07 | -.26 | .02 | .09 | .07 | -.05 | .23 | .04 | .07 | -.10 | .19 |
| SI x Binding | .05 | .07 | -.08 | .19 | -.15** | .06 | -.27 | -.04 | -.01 | .06 | -.13 | .12 |
| *R^2^_adj_* | .23 |  |  |  | .46 |  |  |  | .32 |  |  |  |
| *ΔR^2^_adj_* | .00 |  |  |  | .04 |  |  |  | .02 |  |  |  |
| Step 3 |  |  |  |  |  |  |  |  |  |  |  |  |
| PI x SI x individualizing | .02 | .05 | -.09 | .12 | .05 | .07 | -.08 | .18 | .05 | .06 | -.07 | .17 |
| PI x SI x binding | .09 | .05 | -.02 | .20 | .04 | .04 | -.04 | .12 | .08 | .05 | -.01 | .18 |
| *R^2^_adj_* | .23 |  |  |  | .46 |  |  |  | .33 |  |  |  |
| *ΔR^2^_adj_* | .00 |  |  |  | .00 |  |  |  | .01 |  |  |  |

*Note*. Analyses with data exclusion criteria applied (*N* = 250; see main text for criteria). PI = personal identity and SI = social identity. LL = lower limit for 95% CI for β; UL = upper limit for 95% CI for β. For the issue of gun control, the PI x SI interaction accounted for 2% of variance in the model, but simple slopes analysis revealed that the slopes at +1 and -1 SD of the social identity measure were not significantly different from zero. The other significant interactions for the issue of gun control did not account for more than 1% of the variance.

**p* < .05. ***p* < .01.

**S10 Table. Goodness-of-Fit Indicators for Confirmatory Factor Analysis Models for Identity Function Measure (Indian Sample)**

| Model | *X^2^* | df | *X^2^* diff | CFI | BIC | RMSEA |
| --- | --- | --- | --- | --- | --- | --- |
| **Fossil Fuels** |  |  |  |  |  |  |
| One-factor | 735.93 | 152 |  | .79 | 7169.39 | .16 |
| Two-factor | 262.91 | 151 | 473.02** | .96 | 6701.38 | .07 |
| **Oil Exploration** |  |  |  |  |  |  |
| One-factor | 844.90 | 152 |  | .76 | 7274.30 | .17 |
| Two-factor | 269.38 | 151 | 575.53** | .96 | 6703.80 | .07 |
| **Plastic Ban** |  |  |  |  |  |  |
| One-factor | 1150.49 | 152 |  | .64 | 7303.43 | .21 |
| Two-factor | 302.42 | 151 | 848.07** | .95 | 6460.38 | .08 |

*Note*. Using BIC, not sample-size adjusted BIC.

**p* < .05. ***p* < .01.

**S11 Table. Goodness-of-Fit Indicators for Confirmatory Factor Analysis Models for Identity Function Measure (American Sample)**

| Model | *X^2^* | df | *X^2^* diff | CFI | BIC | RMSEA |
| --- | --- | --- | --- | --- | --- | --- |
| **Fossil Fuels** |  |  |  |  |  |  |
| One-factor | 1529.23 | 152 |  | .61 | 7457.68 | .24 |
| Two-factor | 556.39 | 151 | 972.84** | .88 | 6489.89 | .13 |
| **Oil Exploration** |  |  |  |  |  |  |
| One-factor | 1367.37 | 152 |  | .69 | 7144.48 | .23 |
| Two-factor | 425.73 | 151 | 941.64** | .93 | 6207.89 | .11 |
| **Plastic Ban** |  |  |  |  |  |  |
| One-factor | 1525.09 | 152 |  | .62 | 7303.81 | .24 |
| Two-factor | 483.26 | 151 | 1041.80** | .91 | 6267.01 | .12 |

*Note*. Using BIC, not sample-size adjusted BIC.

**p* < .05. ***p* < .01.

**S12 Table. Standardized Factor Loadings for 2-factor Confirmatory Model for Identity Function Measure for Issues of Fossil Fuels, Oil Exploration, and Plastic Ban in Study 3 (Indian Sample)**

|  | FF | OE | PB | FF | OE | PB |
| --- | --- | --- | --- | --- | --- | --- |
|  | Factor 1: PI |  |  | Factor 2: SI |  |  |
| My sense of who I am as a person | .88 | .86 | .84 |  |  |  |
| My true self | .90 | .88 | .88 |  |  |  |
| The real me | .91 | .90 | .86 |  |  |  |
| My core self | .87 | .85 | .82 |  |  |  |
| Who I am as a person | .86 | .90 | .89 |  |  |  |
| My own personal well-being and self-esteem | .74 | .80 | .83 |  |  |  |
| Important part of who I am | .81 | .85 | .86 |  |  |  |
| My ideas about what kind of person I really am | .83 | .86 | .80 |  |  |  |
| My private opinions of myself | .72 | .78 | .75 |  |  |  |
| My internal guiding principles | .79 | .83 | .77 |  |  |  |
| My desire to maintain close relationships |  |  |  | .85 | .91 | .88 |
| My desire to avoid unnecessary conflict with others |  |  |  | .79 | .79 | .75 |
| A signal to others that I am a good group member |  |  |  | .83 | .87 | .85 |
| Values of the group of people most important to me |  |  |  | .86 | .79 | .83 |
| My identification with central groups in my life |  |  |  | .85 | .82 | .91 |
| My desire to avoid being rejected by others who are important to me |  |  |  | .79 | .86 | .78 |
| Reputation (what others think of me) |  |  |  | .81 | .80 | .81 |
| Feelings of connectedness with those who I am close to |  |  |  | .89 | .90 | .85 |
| Relationships with those I feel close to |  |  |  | .91 | .88 | .91 |

*Note*. *N* = 153 (Complete pairwise observations). FF = fossil fuels, OE = oil exploration, PB = plastic ban.

**S13 Table. Standardized Factor Loadings for 2-factor Confirmatory Model for Identity Function Measure for Issues of Fossil Fuels, Oil Exploration, and Plastic Ban in Study 3 (American Sample)**

|  | FF | OE | PB | FF | OE | PB |
| --- | --- | --- | --- | --- | --- | --- |
|  | Factor 1: PI |  |  | Factor 2: SI |  |  |
| My sense of who I am as a person | .94 | .95 | .90 |  |  |  |
| My true self | .94 | .95 | .95 |  |  |  |
| The real me | .91 | .97 | .93 |  |  |  |
| My core self | .94 | .95 | .95 |  |  |  |
| Who I am as a person | .94 | .97 | .94 |  |  |  |
| My own personal well-being and self-esteem | .79 | .81 | .81 |  |  |  |
| Important part of who I am | .85 | .92 | .93 |  |  |  |
| My ideas about what kind of person I really am | .93 | .93 | .88 |  |  |  |
| My private opinions of myself | .84 | .88 | .81 |  |  |  |
| My internal guiding principles | .83 | .90 | .90 |  |  |  |
| My desire to maintain close relationships |  |  |  | .85 | .81 | .88 |
| My desire to avoid unnecessary conflict with others |  |  |  | .67 | .68 | .75 |
| A signal to others that I am a good group member |  |  |  | .86 | .88 | .85 |
| Values of the group of people most important to me |  |  |  | .83 | .79 | .77 |
| My identification with central groups in my life |  |  |  | .86 | .86 | .85 |
| My desire to avoid being rejected by others who are important to me |  |  |  | .73 | .75 | .76 |
| Reputation (what others think of me) |  |  |  | .73 | .78 | .84 |
| Feelings of connectedness with those who I am close to |  |  |  | .84 | .89 | .88 |
| Relationships with those I feel close to |  |  |  | .88 | .84 | .91 |

*Note*. *N* = 161 (Complete pairwise observations). FF = fossil fuels, OE = oil exploration, PB = plastic ban.

**S14 Table.** **Descriptive Statistics and Correlations for Issue of Fossil Fuels with Covariates**

| *Variable* | *M* | *SD* | 1 | 2 | 3 | 4 | 5 | 6 |
| --- | --- | --- | --- | --- | --- | --- | --- | --- |
| 1. Moral conviction | 3.44 | 1.18 |  |  |  |  |  |  |
| 2. Personal identity | 3.46 | 1.09 | .78** |  |  |  |  |  |
| 3. Social identity | 2.57 | 1.22 | .47** | .55** |  |  |  |  |
| 4. Attitude certainty | 3.66 | 1.12 | .71** | .66** | .36** |  |  |  |
| 5. Attitude importance | 3.55 | 1.05 | .73** | .69** | .49** | .74** |  |  |
| 6. Issue position | 1.87 | 3.22 | .34** | .32** | .21** | .20** | .22** |  |
| 7. Country |  |  | .20** | .26** | .57** | .17** | .25** | .09 |

*Note*. *N* = 300 (complete pairwise observations). Country (U.S. = 0, India = 1).

**p* < .05. ***p* < .01.

**S15 Table*.*** **Descriptive Statistics and Correlations for Issue of Oil Exploration** **with Covariates**

| *Variable* | *M* | *SD* | 1 | 2 | 3 | 4 | 5 |  |
| --- | --- | --- | --- | --- | --- | --- | --- | --- |
| 1. Moral conviction | 3.24 | 1.26 |  |  |  |  |  |  |
| 2. Personal identity | 3.33 | 1.19 | .80** |  |  |  |  |  |
| 3. Social identity | 2.52 | 1.21 | .44** | .54** |  |  |  |  |
| 4. Attitude certainty | 3.37 | 1.26 | .81** | .74** | .42** |  |  |  |
| 5. Attitude importance | 3.30 | 1.22 | .82** | .79** | .49** | .82** |  |  |
| 6. Issue position | -0.23 | 3.47 | -.12* | -.03 | .37** | -.02 | -.06 |  |
| 7. Country |  |  | .12* | .20** | .56** | .18** | .16** | .42** |

*Note*. *N* = 300 (complete pairwise observations). Country (U.S. = 0, India = 1).

**p* < .05. ***p* < .01.

**S16 Table. Descriptive Statistics and Correlations for Issue of Plastic Ban with Covariates**

| *Variable* | *M* | *SD* | 1 | 2 | 3 | 4 | 5 |  |
| --- | --- | --- | --- | --- | --- | --- | --- | --- |
| 1. Moral conviction | 3.59 | 1.23 |  |  |  |  |  |  |
| 2. Personal identity | 3.61 | 1.10 | .82** |  |  |  |  |  |
| 3. Social identity | 2.68 | 1.31 | .49** | .55** |  |  |  |  |
| 4. Attitude certainty | 3.86 | 1.12 | .73** | .69** | .41** |  |  |  |
| 5. Attitude importance | 3.72 | 1.14 | .78** | .75** | .45** | .79** |  |  |
| 6. Issue position | 3.33 | 2.37 | .59** | .61** | .41** | .54** | .64** |  |
| 7. Country |  |  | .38** | .40** | .63** | .39** | .46** | .44** |

*Note*. *N* = 300 (complete pairwise observations). Country (U.S. = 0, India = 1).

**p* < .05. ***p* < .01.

**S17 Table. Descriptive Statistics and Correlations for Issue of Fossil Fuels in Study 3 (American sample)**

| *Variable* | *M* | *SD* | 1 | 2 | 3 | 4 | 5 |
| --- | --- | --- | --- | --- | --- | --- | --- |
| 1. Moral conviction | 3.19 | 1.28 |  |  |  |  |  |
| 2. Personal identity | 3.18 | 1.18 | .79** |  |  |  |  |
| 3. Social identity | 1.88 | 0.89 | .34** | .33** |  |  |  |
| 4. Attitude certainty | 3.47 | 1.27 | .65** | .60** | .20* |  |  |
| 5. Attitude importance | 3.29 | 1.11 | .65** | .61** | .27** | .69** |  |
| 6. Issue position | 1.59 | 3.24 | .41** | .30** | .15 | .18* | .19* |

*Note*. *N* = 150 (complete pairwise observations).

**p* < .05. ***p* < .01.

**S18 Table. Descriptive Statistics and Correlations for Issue of Oil Exploration in Study 3 (American sample)**

| *Variable* | *M* | *SD* | 1 | 2 | 3 | 4 | 5 |
| --- | --- | --- | --- | --- | --- | --- | --- |
| 1. Moral conviction | 3.09 | 1.44 |  |  |  |  |  |
| 2. Personal identity | 3.09 | 1.31 | .83** |  |  |  |  |
| 3. Social identity | 1.85 | 0.91 | .36** | .39** |  |  |  |
| 4. Attitude certainty | 3.14 | 1.42 | .81** | .75** | .28** |  |  |
| 5. Attitude importance | 3.10 | 1.37 | .85** | .83** | .33** | .86** |  |
| 6. Issue position | -1.70 | 3.11 | -.52** | -.43** | -.07 | -.37** | -.41** |

*Note*. *N* = 150 (complete pairwise observations).

**p* < .05. ***p* < .01.

**S19 Table. Descriptive Statistics and Correlations for Issue of Plastic Ban in Study 3 (American sample)**

| *Variable* | *M* | *SD* | 1 | 2 | 3 | 4 | 5 |
| --- | --- | --- | --- | --- | --- | --- | --- |
| 1. Moral conviction | 3.12 | 1.38 |  |  |  |  |  |
| 2. Personal identity | 3.18 | 1.18 | .82** |  |  |  |  |
| 3. Social identity | 1.85 | 0.94 | .32** | .37** |  |  |  |
| 4. Attitude certainty | 3.42 | 1.25 | .70** | .64** | .23** |  |  |
| 5. Attitude importance | 3.19 | 1.23 | .78** | .73** | .24** | .77** |  |
| 6. Issue position | 2.29 | 2.70 | .53** | .56** | .19* | .42** | .57** |

*Note*. *N* = 150 (complete pairwise observations).

**p* < .05. ***p* < .01.

**S20 Table. Descriptive Statistics and Correlations for Issue of Fossil Fuels in Study 3 (Indian sample)**

| *Variable* | *M* | *SD* | 1 | 2 | 3 | 4 | 5 |
| --- | --- | --- | --- | --- | --- | --- | --- |
| 1. Moral conviction | 3.68 | 1.02 |  |  |  |  |  |
| 2. Personal identity | 3.74 | 0.92 | .72** |  |  |  |  |
| 3. Social identity | 3.27 | 1.10 | .57** | .72** |  |  |  |
| 4. Attitude certainty | 3.85 | 0.92 | .78** | .72** | .49** |  |  |
| 5. Attitude importance | 3.82 | 0.91 | .81** | .77** | .60** | .81** |  |
| 6. Issue position | 2.15 | 3.18 | .23** | .31** | .25** | .21* | .23** |

*Note*. *N* = 150 (complete pairwise observations).

**p* < .05. ***p* < .01.

**S21 Table. Descriptive Statistics and Correlations for Issue of Oil Exploration in Study 3 (Indian sample)**

| *Variable* | *M* | *SD* | 1 | 2 | 3 | 4 | 5 |
| --- | --- | --- | --- | --- | --- | --- | --- |
| 1. Moral conviction | 3.39 | 1.03 |  |  |  |  |  |
| 2. Personal identity | 3.57 | 1.02 | .73** |  |  |  |  |
| 3. Social identity | 3.20 | 1.10 | .60** | .70** |  |  |  |
| 4. Attitude certainty | 3.59 | 1.04 | .80** | .71** | .54** |  |  |
| 5. Attitude importance | 3.49 | 1.02 | .75** | .70** | .69** | .72** |  |
| 6. Issue position | 1.24 | 3.17 | .25** | .26** | .39** | .24* | .19* |

*Note*. *N* = 150 (complete pairwise observations).

**p* < .05. ***p* < .01.

**S22 Table. Descriptive Statistics and Correlations for Issue of Plastic Ban in Study 3 (Indian sample)**

| *Variable* | *M* | *SD* | 1 | 2 | 3 | 4 | 5 |
| --- | --- | --- | --- | --- | --- | --- | --- |
| 1. Moral conviction | 4.06 | 0.85 |  |  |  |  |  |
| 2. Personal identity | 4.05 | 0.81 | .70** |  |  |  |  |
| 3. Social identity | 3.50 | 1.10 | .44** | .51** |  |  |  |
| 4. Attitude certainty | 4.30 | 0.76 | .63** | .61** | .26** |  |  |
| 5. Attitude importance | 4.24 | 0.75 | .62** | .61** | .27** | .67** |  |
| 6. Issue position | 4.37 | 1.34 | .46** | .42** | .22* | .53** | .50** |

*Note*. *N* = 150 (complete pairwise observations).

**p* < .05. ***p* < .01.

**S23 Table. Hierarchical Regression Results (Standardized Coefficients) for Predicting Moral Conviction Across Issues Controlling for Covariates in Study 3**

|  | *Fossil Fuels* |  |  |  | *Oil Exploration* |  |  |  | *Plastic Ban* |  |  |  |
| --- | --- | --- | --- | --- | --- | --- | --- | --- | --- | --- | --- | --- |
| Predictor | *β* | *SE β* | *LL* | *UL* | *β* | *SE β* | *LL* | *UL* | *β* | *SE β* | *LL* | *UL* |
| Step 1 |  |  |  |  |  |  |  |  |  |  |  |  |
| Attitude importance | .42** | .05 | .31 | .52 | .47** | .05 | .37 | .57 | .46** | .06 | .34 | .58 |
| Attitude certainty | .35** | .05 | .26 | .47 | .43** | .05 | .32 | .53 | .29** | .06 | .18 | .40 |
| Issue position | .17** | .04 | .10 | .25 | -.08* | .03 | -.14 | -.02 | .13** | .04 | .05 | .22 |
| *R^2^_adj_* | .62 |  |  |  | .74 |  |  |  | .65 |  |  |  |
| Step 2 |  |  |  |  |  |  |  |  |  |  |  |  |
| Personal identity | .42** | .05 | .32 | .52 | .30** | .05 | .20 | .40 | .46** | .05 | .37 | .56 |
| Social identity | .03 | .04 | -.05 | .10 | -.04 | .04 | -.04 | .11 | .03 | .04 | -.04 | .10 |
| *R^2^_adj_* | .70 |  |  |  | .77 |  |  |  | .74 |  |  |  |
| *ΔR^2^_adj_* | .08 |  |  |  | .03 |  |  |  | .09 |  |  |  |
| Step 3 |  |  |  |  |  |  |  |  |  |  |  |  |
| Country | -.10 | .08 | -.25 | .06 | -.09 | .07 | -.23 | .05 | -.10 | .08 | -.25 | .06 |
| Personal identity*Social identity | -.03 | .04 | -.12 | .05 | -.01 | .04 | -.09 | .07 | -.03 | .05 | -.13 | .07 |
| PI x Country | -.16 | .09 | -.34 | .01 | -.06 | .08 | -.22 | .10 | -.15 | .09 | -.32 | .03 |
| SI x Country | .01 | .10 | -.18 | .20 | -.01 | .09 | -.18 | .16 | .07 | .09 | -.10 | .24 |
| *R^2^_adj_* | .71 |  |  |  | .77 |  |  |  | .74 |  |  |  |
| *ΔR^2^_adj_* | .01 |  |  |  | .00 |  |  |  | .00 |  |  |  |
| Step 4 |  |  |  |  |  |  |  |  |  |  |  |  |
| PI x SI x Country | .11 | .09 | -.06 | .28 | -.04 | .08 | -.21 | .12 | -.04 | .10 | -.23 | .16 |
| *R^2^_adj_* | .70 |  |  |  | .77 |  |  |  | .74 |  |  |  |
| *ΔR^2^_adj_* | -.01 |  |  |  | .00 |  |  |  | .00 |  |  |  |

*Note*. *N* = 300. PI = personal identity; SI = social identity; LL = lower limit for 95% CI for β; UL = upper limit for 95% CI for β.

**p* < .05. ***p* < .01.

**S24 Table. Hierarchical Regression Results (Standardized Coefficients) for Predicting Moral Conviction Across Issues Without Covariates in Study 3**

|  | *Fossil Fuels* |  |  |  | *Oil Exploration* |  |  |  | *Plastic Ban* |  |  |  |
| --- | --- | --- | --- | --- | --- | --- | --- | --- | --- | --- | --- | --- |
| Predictor | *β* | *SE β* | *LL* | *UL* | *β* | *SE β* | *LL* | *UL* | *β* | *SE β* | *LL* | *UL* |
| Step 1 |  |  |  |  |  |  |  |  |  |  |  |  |
| Personal identity | .74** | .04 | .66 | .83 | .78** | .04 | .70 | .87 | .78** | .04 | .70 | .86 |
| Social identity | .06 | .05 | -.03 | .15 | .02 | .04 | -.06 | .11 | .06 | .04 | -.02 | .14 |
| *R^2^_adj_* | .61 |  |  |  | .63 |  |  |  | .67 |  |  |  |
| Step 2 |  |  |  |  |  |  |  |  |  |  |  |  |
| Country | -.07 | .09 | -.25 | .11 | -.18* | .09 | -.36 | -.01 | .13 | .09 | -.04 | .30 |
| Personal identity*Social identity | .02 | .05 | -.08 | .12 | -.06 | .05 | -.16 | .05 | .04 | .05 | -.06 | .15 |
| PI x Country | -.13 | .11 | -.34 | .08 | -.26* | .10 | -.46 | -.07 | -.27* | .10 | -.47 | -.08 |
| SI x Country | <-.01 | .11 | -.23 | .22 | .11 | .11 | -.10 | .32 | .03 | .10 | -.16 | .22 |
| *R^2^_adj_* | .60 |  |  |  | .64 |  |  |  | .68 |  |  |  |
| *ΔR^2^_adj_* | -.01 |  |  |  | .01 |  |  |  | .01 |  |  |  |
| Step 3 |  |  |  |  |  |  |  |  |  |  |  |  |
| PI x SI x Country | -.02 | .10 | -.22 | .19 | -.17 | .11 | -.38 | .04 | -.15 | .11 | -.37 | .06 |
| *R^2^_adj_* | .60 |  |  |  | .64 |  |  |  | .68 |  |  |  |
| *ΔR^2^_adj_* | .00 |  |  |  | .00 |  |  |  | .00 |  |  |  |

*Note*. Analyses with data exclusion criteria applied (i.e., participants who took less than 3 minutes to complete the study (*N* = 15) or did not pass at least 1 of the 2 attention checks (*N* = 8); Final *N* = 283). PI = personal identity; SI = social identity; LL = lower limit for 95% CI for β; UL = upper limit for 95% CI for β. None of the significant interactions accounted for more than 1% of the variance in the model.

**p* < .05. ***p* < .01.

**S25 Table. Hierarchical Regression Results (Standardized Coefficients) Examining Unique Effect of Personal Identity Function on Moral Conviction Across Issues Controlling for Covariates in Study 1**

|  | *Same-sex marriage* |  |  |  | *Gun control* |  |  |  | *Capital punishment* |  |  |  |
| --- | --- | --- | --- | --- | --- | --- | --- | --- | --- | --- | --- | --- |
| Predictor | *β* | *SE β* | *LL* | *UL* | *β* | *SE β* | *LL* | *UL* | *β* | *SE β* | *LL* | *UL* |
| Step 1 |  |  |  |  |  |  |  |  |  |  |  |  |
| Attitude importance | .33** | .05 | .24 | .42 | .55** | .05 | .44 | .66 | .49** | .06 | .37 | .60 |
| Attitude certainty | .60** | .05 | .51 | .69 | .26** | .05 | .16 | .37 | .35** | .05 | .24 | .46 |
| Issue position | -.17** | .04 | -.24 | -.10 | <-.01 | .04 | -.07 | .07 | -.01 | .04 | -.08 | .06 |
| Social identity | .04 | .04 | -.04 | 0.11 | .06 | .04 | -.02 | .13 | -.03 | .04 | -.11 | .06 |
| *R^2^_adj_* | .67 |  |  |  | .63 |  |  |  | .59 |  |  |  |
| Step 2 |  |  |  |  |  |  |  |  |  |  |  |  |
| Personal identity | .18** | .05 | .08 | .28 | .18** | .05 | .07 | .28 | .27** | .05 | .17 | .37 |
| *R^2^_adj_* | .68 |  |  |  | .64 |  |  |  | .62 |  |  |  |
| *ΔR^2^_adj_* | .01 |  |  |  | .01 |  |  |  | .03 |  |  |  |

*Note*. *N* = 320. LL = lower limit for 95% CI for β; UL = upper limit for 95% CI for β.

**p* < .05. ***p* < .01

**S26 Table. Hierarchical Regression Results (Standardized Coefficients) Examining Unique Effect of Personal Identity Function on Moral Conviction Across Issues Controlling for Covariates in Study 2**

|  | *Same-sex marriage* |  |  |  | *Gun control* |  |  |  | *Capital punishment* |  |  |  |
| --- | --- | --- | --- | --- | --- | --- | --- | --- | --- | --- | --- | --- |
| Predictor | *β* | *SE β* | *LL* | *UL* | *β* | *SE β* | *LL* | *UL* | *β* | *SE β* | *LL* | *UL* |
| Step 1 |  |  |  |  |  |  |  |  |  |  |  |  |
| Attitude importance | .24** | .05 | .14 | .34 | .42** | .05 | .32 | .52 | .27** | .06 | .16 | .39 |
| Attitude certainty | .58** | .04 | .49 | .66 | .32** | .05 | .22 | .41 | .38** | .05 | .27 | .48 |
| Issue position | -.16** | .04 | -.24 | -.09 | -.08* | .04 | -.15 | -.01 | -.08 | .04 | -.17 | <-.01 |
| Social identity | .05 | .04 | -.03 | .14 | .09* | .04 | .01 | .17 | .04 | .05 | -.05 | .13 |
| *R^2^_adj_* | .52 |  |  |  | .55 |  |  |  | .37 |  |  |  |
| Step 2 |  |  |  |  |  |  |  |  |  |  |  |  |
| Personal identity | .17** | .05 | .08 | .27 | .34** | .05 | .24 | .43 | .32** | .06 | .21 | .43 |
| *R^2^_adj_* | .54 |  |  |  | .60 |  |  |  | .42 |  |  |  |
| *ΔR^2^_adj_* | .02 |  |  |  | .05 |  |  |  | .05 |  |  |  |

*Note*. *N* = 364. LL = lower limit for 95% CI for β; UL = upper limit for 95% CI for β.

**p* < .05. ***p* < .01

**S27 Table. Hierarchical Regression Results (Standardized Coefficients) Examining Unique Effect of Personal Identity Function on Moral Conviction Across Issues Controlling for Covariates in Study 3**

|  | *Fossil Fuels* |  |  |  | *Oil Exploration* |  |  |  | *Plastic Ban* |  |  |  |
| --- | --- | --- | --- | --- | --- | --- | --- | --- | --- | --- | --- | --- |
| Predictor | *β* | *SE β* | *LL* | *UL* | *β* | *SE β* | *LL* | *UL* | *β* | *SE β* | *LL* | *UL* |
| Step 1 |  |  |  |  |  |  |  |  |  |  |  |  |
| Attitude importance | .35** | .06 | .24 | .47 | .41** | .05 | .31 | .52 | .43** | .06 | .31 | .55 |
| Attitude certainty | .37** | .05 | .26 | .47 | .43** | .05 | .33 | .53 | .27** | .05 | .16 | .38 |
| Issue position | .16** | .04 | .09 | .23 | -.12* | .03 | -.19 | -.06 | .11* | .04 | .03 | .20 |
| Social identity | .14** | .04 | .06 | .12 | .11** | .0 | .03 | .18 | .14** | .04 | .06 | .21 |
| *R^2^_adj_* | .63 |  |  |  | .74 |  |  |  | .66 |  |  |  |
| Step 2 |  |  |  |  |  |  |  |  |  |  |  |  |
| Personal identity | .42** | .05 | .32 | .52 | .30** | .05 | .21 | .40 | .46** | .05 | .37 | .56 |
| *R^2^_adj_* | .70 |  |  |  | .77 |  |  |  | .74 |  |  |  |
| *ΔR^2^_adj_* | .07 |  |  |  | .03 |  |  |  | .08 |  |  |  |

*Note*. *N* = 300. LL = lower limit for 95% CI for β; UL = upper limit for 95% CI for β.

**p* < .05. ***p* < .01

**S1 Appendix**

**Pilot Test for Study 3**

To account for potential differences in the current (hot button) issues of the day in India versus the United States and additionally test the robustness of the results from previous studies, a pilot study was conducted to select three new issues for Study 3. The issues were selected with the assistance of someone with deep cultural knowledge of both the U.S. and India. Our goal was to identify three issues that yielded the highest reliability indices (Cronbach’s alpha) and similar distributions of moral conviction across both samples/cultures. We also looked at the distributions of attitude importance for each issue.

**Method**

**Participants**

Sixty-five Indian CloudResearch participants and 66 US CloudResearch participants completed the study. Participants were recruited on December 28, 2020. Data cleaning involved removing preview/test responses (*N* = 2 for the Indian sample and *N* = 4 for American sample) and removing duplicate IDs (including blanks where participants had incomplete responses; *N* = 4 for Indian sample and *N* = 5 for American sample), which produced the final sample of *N* = 65 Indian participants and *N* = 66 American participants. All participants passed the data quality check.

**Procedure**

Participants were asked to provide attitude ratings for six different issues. For each issue, they provided information on their position on the topic, the strength of their position, and attitude importance and moral conviction for each issue. The issue blocks were randomized. The wording of the six issues was the following: “higher taxes on fossil fuels in an effort to reduce greenhouse gases and climate change”, “lifting restrictions on future oil exploration and drilling”, “higher taxes on the wealthy to reduce income inequality”, “government regulations designed to reduce the distribution of fake news on social media”, “laws that prohibit discrimination based on gender in the workplace”, and “banning the use of plastic bags, straws, and cutlery to help protect the environment”.

**Measures**

***Issue Position***

To obtain the issue position (stance) a participant had for each issue, participants received the question “Do you support or oppose [ISSUE]?” and then responded on a 3-point scale with point labels of *support, neutral/uncertain,* and *oppose*. Participants then provided information about the strength of their issue position by responding to the question “How strongly do you support[/oppose] [ISSUE]?” if they had selected *support* or *oppose* to the issue position question. Participants responded on a 4-point scale with point labels of *slightly, moderately, much*, and *very much* for this question. If participants indicated a response choice of *neutral/uncertain* in the issue position question, they branched to the question “Do you lean towards supporting or opposing [ISSUE]?” and gave their rating on a 3-point scale with point labels of *lean towards supporting, neutral,* and *lean towards opposing*. These items were combined to form a single bi-polar scale from -5 *strongly oppose* to 5 *strongly support* (with the neutral point as 0).

***Attitude Strength (Moral Conviction and Importance)***

Participants provided information about attitude strength indicators of moral conviction and importance using two items for each. They received the statement “To what extent is your position on [ISSUE]…” and then for moral conviction, they received the following stems: “Connected to your beliefs about fundamental right and wrong?” and “a reflection of your core moral beliefs and convictions?”. For attitude importance, the stems were “something that you care a lot about?” and “personally important to you?”. Participants gave their responses using a 5-point scale with point labels of *not at all, slightly, moderately, much,* and *very much.* The two items for moral conviction were averaged to create a single score, and the same was done for importance.

***Data quality check***

One data quality check was included in the study that was a surface-level way of assessing English comprehension. Participants were given a fill-in-the-blank question of “What is your favorite color?” and wrote in their response. Participants who provided a valid color name were counted as high quality responses.

**Pilot Study Results**

Descriptive analyses were conducted for moral conviction, attitude importance, and issue position for each of the issues, as well as reliability analyses for moral conviction and attitude importance (see Tables A-C for details). To select the issues for Study 3, we prioritized examination of the moral conviction variable for each sample (US and Indian). Reliability indices for moral conviction were lower in the Indian than the US sample, but in an acceptable range for a two-item measure. The issues across the two samples that had the best reliability for moral conviction *and* similar moral conviction ratings were fossil fuels, oil exploration, and the plastic ban.

**Table A. Descriptive Statistics for the Variable of Moral Conviction**

| *Issue* | American |  |  | Indian |  |  |
| --- | --- | --- | --- | --- | --- | --- |
|  | *M* | *SD* | *α* | *M* | *SD* | *α* |
| Fossil fuels | 3.47 | 1.26 | .94 | 3.52 | 0.78 | .64 |
| Oil exploration | 3.10 | 1.31 | .95 | 3.59 | 0.81 | .70 |
| Income inequality | 3.71 | 1.16 | .90 | 3.61 | 0.76 | .57 |
| Fake news | 3.32 | 1.18 | .95 | 3.65 | 0.74 | .50 |
| Gender discrimination | 4.28 | 0.96 | .93 | 3.72 | 0.80 | .64 |
| Plastic ban | 3.05 | 1.30 | .91 | 3.73 | 0.80 | .68 |

*Note. N* = 65 (using complete pairwise observations).

**Table B. Descriptives Statistics for the Variable of Attitude Importance**

| *Issue* | American |  |  | Indian |  |  | |
| --- | --- | --- | --- | --- | --- | --- | --- |
|  | *M* | *SD* | *α* | *M* | *SD* | | *α* |
| Fossil fuels | 3.41 | 1.20 | .96 | 3.72 | 0.73 | | .62 |
| Oil exploration | 2.98 | 1.26 | .96 | 3.71 | 0.80 | | .65 |
| Income inequality | 3.59 | 1.28 | .97 | 3.68 | 0.83 | | .76 |
| Fake news | 3.34 | 1.20 | .95 | 3.69 | 0.73 | | .62 |
| Gender discrimination | 3.80 | 1.32 | .93 | 3.73 | 0.72 | | .52 |
| Plastic ban | 3.21 | 1.24 | .94 | 3.72 | 0.71 | | .54 |

*Note. N* = 65 (using complete pairwise observations).

**Table C. Descriptives Statistics for the Variable of Issue Position**

| *Issue* | American |  | Indian |  |
| --- | --- | --- | --- | --- |
|  | *M* | *SD* | *M* | *SD* |
| Fossil Fuels | 1.92 | 3.29 | 2.09 | 2.25 |
| Oil Exploration | -0.95 | 3.44 | 2.14 | 2.12 |
| Income inequality | 2.68 | 2.82 | 2.29 | 2.27 |
| Fake news | 1.29 | 3.27 | 2.38 | 2.03 |
| Gender Discrimination | 3.91 | 1.77 | 2.28 | 2.08 |
| Plastic ban | 1.60 | 3.22 | 1.98 | 2.72 |

*Note. N* = 65 (using complete pairwise observations).

**S2 Appendix**

**Screening for Study 3 participation**

All study materials were in English. Although knowledge of English is very common in India, we wanted to ensure that differences in language comprehension would not be a confound across our U.S. and Indian samples. All participants therefore had to complete an English comprehension test before proceeding to the main part of Study 3.

**Screener Method**

**Participants and procedure**

Four hundred seventy-five Indian participants and 246 US participants were recruited to participate in a brief survey to see if they qualified for the main study. In the posting for this survey, we specifically asked people to sign up who were fluent in English. Given that participants for the main study would be selected from these screener samples, we recruited a larger number of participants than we would need to account for people not passing the screener and for attrition between the screener and the main study (which we estimated as approximately 15%). We initially recruited 201 Indian and 201 US participants on January 5, 2021, with the aim to have 150 participants for each country in our main study based on an a priori power analysis. However, we needed to recruit more participants in the Indian sample because only about 39% passed the screener on the first round of data collection compared to 90% passing in the US sample. Thus, 275 more Indian participants and 45 more US participants were recruited (with Indian participants recruited from January 14, 2021 to January 15, 2021 and American participants on February 1, 2021), to make sure that we would have at least 200 participants from each country for the main study.

**Measure of English Comprehension**

An English comprehension screener was taken from a book of reading comprehension questions (501 Reading Comprehension Questions, 2010). Participants were given the following prompt to read: “In criminal cases, the availability of readable fingerprints is often critical in establishing evidence of a major crime. It is necessary, therefore, to follow proper procedures when taking fingerprints. In major cases, prints should be obtained from all persons who may have touched areas associated with a crime scene, for elimination purposes.” After reading the prompt, they answered the question, “The paragraph best supports the statement that” with one of four possible answer choices (with the bold response representing the correct answer):

No crimes can be solved without readable fingerprints.

All persons who have touched an area in a crime scene are suspects.

All fingerprints found at a crime scene are used in court as evidence.

**All persons who have touched a crime-scene area should be fingerprinted.**

**Results**

After examining the responses to the screener, 219 American participants and 265 Indian participants were eligible to be contacted to participate in the main study.

**References**

501 Reading Comprehension Questions. (2010). 4^th^ edition. LearningExpress, New York: NY.
